# Dynamic phosphorylation of DGCR8 regulates embryonic stem cell differentiation and microprocessor function

**DOI:** 10.3389/fcell.2026.1918038

**Published:** 2026-10-01

**Authors:** Ka Weng Leong, Lawrence Mok, Karen Gu, Jonathan S. Oakhill, Mark M. W. Chong

**Affiliations:** 1 St Vincent’s Institute of Medical Research, Fitzroy, VIC, Australia; 2 Department of Medicine (St Vincent’s), University of Melbourne, Fitzroy, VIC, Australia

**Keywords:** DGCR8, DROSHA, microprocessor, microRNA pathway, phosphorylation, stem cell differentiation

## Abstract

The Microprocessor complex, minimally composed of DROSHA and DGCR8, is ubiquitously required for microRNA biogenesis. It also regulates the balance between pluripotency and differentiation through mRNA alternative splicing and mRNA cleavage. However, how these various activities are regulated remains incompletely understood. Both DROSHA and DGCR8 are phosphoproteins, and phosphorylation has been reported to influence their molecular functions. In this study, we identified residues in DGCR8 by mass spectrometry that were differentially phosphorylated in mouse embryonic stem cells (mESCs) before and after differentiation into neuronal cells. This included S95 that exhibited greater phosphorylation in undifferentiated cells, and S238 that exhibited greater phosphorylation in differentiated cells. By means of CRISPR/Cas9-mediated genome editing of the endogenous *Dgcr8* gene, we generated a homozygous S95A mESC clone and a heterozygous S238A/- mESC clone. Both mutants retained an mESC phenotype but exhibited altered Microprocessor-dependent functions and differentiation outcomes. When directed toward the neuronal lineage, the S238A/- mutant displayed elevated mRNA expression of neuronal progenitor markers even after 10 days, while both S95A and S238A/- mutants failed to generate cardiomyocytes in embryoid body differentiation cultures. Furthermore, MEK1/2 inhibition with a highly selective inhibitor partially phenocopied the transcriptional effects of S95A in mESCs, consistent with a potential contribution of ERK/MAPK signaling for S95 phosphorylation. Together, these findings support a role for site-specific DGCR8 phosphorylation in modulating Microprocessor-dependent processes and differentiation in mESCs, establishing DGCR8 phosphorylation as an important regulatory mechanism in the regulation of Microprocessor activity, especially for stem cell differentiation.

## Introduction

The Microprocessor complex is synonymous with the biogenesis of microRNAs (miRNAs). Its core is composed of the ribonuclease III protein DROSHA and a dimer of a double-stranded RNA-binding protein (dsRBP) DGCR8 (Nguyen et al., 2015). It is responsible for the first processing step in miRNA biogenesis by recognizing and processing the stem-loop structures embedded within primary (pri-)miRNA transcripts (Morlando et al., 2008). The majority of known miRNAs contain sequence and structural motifs to facilitate their recognition (Auyeung et al., 2013; Conrad et al., 2014; Fang and Bartel, 2015; Jin et al., 2020; Li et al., 2021; 2020). DROSHA has low affinity and specificity for its substrate and requires DGCR8 to position the substrate in the correct orientation for productive pri-miRNA processing (Nguyen et al., 2015). The resulting precursor (pre-)miRNA intermediate is subsequently exported to the cytoplasm and further processed by another ribonuclease III protein DICER and its dsRBP cofactor TRBP (Lee et al., 2002; Wilson et al., 2015). Ultimately, a single mature miRNA strand remains and is loaded into the Argonaute-containing RNA-induced silencing complex (RISC), where it mediates translational repression or mRNA degradation (Bartel, 2018). A single mature miRNA can target multiple genes, and most protein-coding transcripts are predicted to be regulated by one or more different miRNAs (Lim et al., 2005; Friedman et al., 2009). Dysregulated miRNA expression is associated with many disorders, including cancer (Garzon et al., 2006; Volinia et al., 2006), immunodeficiency (Jeker et al., 2013), autoimmunity (Chen et al., 2016), and developmental defects (Bartel, 2018).

In addition to pri-miRNAs, the Microprocessor also recognizes other stem-loop containing RNA species for the regulation of diverse biological processes. For example, DROSHA binds stem-loop structures within viral RNAs and inhibits viral RNA-dependent RNA polymerases, thereby contributing to antiviral defense (Aguado et al., 2017). DROSHA also recognizes and cleaves stem-loop structures within protein-coding mRNAs. For example, the 5′ UTR of *DGCR8* mRNA contains two stem-loop structures that are cleaved, which regulates *DGCR8* mRNA turnover (Han et al., 2009). DROSHA binding can also regulate mRNA splicing. It has been shown to bind stem-loop structures within exon five of *eIF4H* pre-mRNA and across the exon seven-intron 7 junction of *DROSHA* pre-mRNA to promote exon skipping (Havens et al., 2014; Lee et al., 2017).

The Microprocessor complex has been reported to regulate stem cell pluripotency and differentiation via miRNA biogenesis (Anokye-Danso et al., 2011; Galagali and Kim, 2020; Ouchi et al., 2013), *Tcf7l1* alternative splicing (Cirera-Salinas et al., 2017), and mRNA cleavage (Johanson et al., 2015; Knuckles et al., 2012). For instance, the expression of the Microprocessor-dependent miRNAs in the *miR302/367* cluster can reprogram somatic cells into pluripotent stem cells (Anokye-Danso et al., 2011). DGCR8 facilitates *Tcf7l1* alternative splicing, thereby promoting exit from pluripotency (Cirera-Salinas et al., 2017). The Microprocessor-mediated cleavage of *Myl9* and *Todr1* mRNAs in hematopoietic stem cells permits myelopoiesis (Johanson et al., 2015), while the cleavage of *Ngn2* and *Nfib* mRNAs in neural stem cells is critical for neurogenesis and oligodendrogenesis, respectively (Knuckles et al., 2012; Rolando et al., 2016).

Post-translational modifications of the miRNA machinery, particularly phosphorylation, have been reported, and these are thought to regulate molecular functions (Ha and Kim, 2014; Paroo et al., 2009). For example, ERK phosphorylates TRBP, enhancing its stability and inducing the production of pro-growth miRNAs (Paroo et al., 2009). Similarly, previous studies reported that DGCR8 phosphorylation can be regulated through the ERK/MAPK pathway and that phosphorylation of multiple DGCR8 residues enhances its stability and promotes the production of pro-growth miRNAs (Herbert et al., 2013). DGCR8 phosphorylation has also been implicated in the regulation of *Tcf7l1* alternative splicing (Cirera-Salinas et al., 2017). Multiple phosphorylation sites have been reported on DROSHA and DGCR8 in high throughput phosphoproteomic studies, as documented in PhosphoSitePlus (Hornbeck et al., 2015; Leong and Chong, 2025). However, the majority of phosphorylation sites identified were based on the analysis of overexpressed tagged proteins in cell lines such as HEK293T (Herbert et al., 2013), and consequently, the physiological and functional relevance of most of these sites remains unclear. In particular, whether dynamic phosphorylation of DGCR8 contributes to the regulation of Microprocessor activity during stem cell differentiation has not been investigated.

In this study, we immunoprecipitated endogenous DROSHA and DGCR8 from mouse embryonic stem cells (mESCs) and neuronal cells derived from mESCs and mapped phospho-sites present on endogenous DROSHA and DGCR8 via mass spectrometry. By estimating the peptide phosphorylation frequencies, we identified DGCR8 residues that were differentially phosphorylated during neuronal differentiation. We employed the CRISPR/Cas9 system to mutate two of the identified sites and investigated the impact of DGCR8 phosphorylation on Microprocessor function and stem cell fate decisions. Our results suggest that the disruption of DGCR8 phosphorylation influences differentiation outcomes and affects Microprocessor-dependent miRNA generation, mRNA cleavage, and alternative splicing.

## Materials and methods

### mESC cultures

E14 mESCs were cultured in Knockout DMEM (Gibco) supplemented with 10% heat-inactivated fetal bovine serum (French origin, Bovogen Biologicals), 5% knockout serum replacement (Gibco), 1× Penicillin-Streptomycin-Glutamine (Gibco), 1× MEM non-essential amino acids (Gibco), 1 mM sodium pyruvate (Gibco), 0.01 mM 2-mercaptoethanol (Sigma-Aldrich), and 500U/mL recombinant mouse LIF (PeproTech) on mitomycin-inactivated mouse embryonic fibroblasts (MEFs). The medium was refreshed every day, and cells were passaged every 2 to 3 days. Prior to harvesting cells for protein or RNA extractions, the MEFs were removed by replating trypsinized single cells onto a few plates for 40min and then harvesting the non-adherent ESCs.

### FDC-P1 cultures

FDC-P1 cells (obtained from ATCC, RRID:CVCL_2039) were maintained in IMDM (Sigma-Aldrich) supplemented with 20% heat-activated horse serum (Sigma-Aldrich), 1× Penicillin-Streptomycin-Glutamine (Sigma-Aldrich), and 5 ng/mL mouse IL-3 (PeproTech). To differentiate FDC-P1 cells into macrophages, mouse IL-3 was withdrawn for 16 h, then 50 ng/mL mouse GM-CSF (PeproTech) was added for 7 days. Flow cytometric analysis for cell size, granularity, and F4/80 expression was performed to confirm myeloid differentiation.

### Neuronal differentiation of mESCs

mESCs were passaged on 0.1% gelatin-coated plates without MEFs for at least three passages prior to differentiation. The cells were then seeded at a density of one–3 × 10^4^/cm^2^ onto 0.1% gelatin-coated plates without LIF for 6 h. The medium was then replaced with N2B27 medium [consisting of 50% DMEM/F12 (Gibco) and 50% Neurobasal medium (Gibco)] supplemented with 1× N2 (Invitrogen) and 1× B27 (Invitrogen), 1× penicillin-streptomycin (Gibco), 1× glutamine (Gibco), and 0.01 mM 2-mercaptoethanol (Sigma-Aldrich). 1µM retinoic acid (Sigma-Aldrich) was added for 48 h. The cells were then cultured in N2B27 medium without retinoic acid (RA) for another 8 days. Medium was refreshed every 2 days.

### Spontaneous differentiation of mESCs

MEF-depleted single cell suspensions of mESCs were seeded in hanging drops at a density of 200 cells/drop, and embryoid bodies (EBs) were allowed to form over 2 days. The formed EBs were then transferred to a 0.1% gelatin-coated well and cultured in mESC medium without LIF for a further 10 days. The medium was refreshed every 2 days.

### Immunoprecipitation

Cells were lysed in RIPA buffer (150 mM NaCl, 1% NP-40, 0.5% sodium deoxycholate, 0.1% SDS, 25 mM Tris-HCl pH 7.4) supplemented with 1 mM PMSF (Sigma-Aldrich), 10 mM NaF, and 1 mM Na_3_VO_4_. Whole cell lysates were incubated with 1/50 vol of anti-DGCR8 antibody (Abcam, ab191875, RRID:AB_2892625), 1/50 vol of anti-DROSHA antibody (Abcam, ab12286, RRID:AB_2301218), or 1/50 volume of Rabbit IgG (Cell Signaling Technology, Cat. #3900, RRID:AB_1550038) overnight at 4 °C. This was followed by incubation with either 1/10 volume of washed Protein G Sepharose beads (Abcam) or Protein G Dynabeads (Invitrogen) for 4 h at 4 °C. Proteins were eluted with 1× LDS (Invitrogen) and 50 mM TCEP (Thermo Fisher Scientific) in RIPA buffer at 100 °C for 10min. Eluted proteins were subjected to immunoblotting, Coomassie Blue or silver staining to confirm the positions of interest.

### Immunoblotting

Proteins were separated on SDS-polyacrylamide gels and transferred onto a PVDF membrane (Amersham). The membranes were blocked with either 5% skim milk or 5% BSA in TBST, followed by overnight incubation at 4 °C with the primary antibodies rabbit anti-DGCR8 (1:1,000, Abcam, ab191875, RRID:AB_2892625), rabbit anti-DROSHA antibody (1:1,000, Abcam, ab12286, RRID:AB_2301218), rabbit anti-β-actin (1:1,000, Cell Signaling Technology, Cat. #4970, RRID:AB_2223172), rabbit anti-Histone H3 (1:1,000, Cell Signaling Technology, Cat. #4499, RRID:AB_10544537), or rabbit anti-phospho-ERK1/2 (1:1,000, Cell Signaling Technology, Cat. #9101, RRID:AB_331646). Goat anti-rabbit-IgG HRP-conjugated secondary antibody (1:5,000, Cell Signaling Technology, Cat #7077, RRID:AB_10694715) and ECL Plus substrate (Thermo Fisher Scientific) or Goat anti-rabbit-IgG DyLight 800 4× PEG-conjugated secondary antibody (1:15,000, Cell Signaling Technology, Cat. #5151, RRID:AB_10697505) were used for visualization.

For ERK1/2 immunoblot, antibodies were stripped off after phospho-ERK1/2 detection with stripping buffer (200 mM glycine, 0.1% SDS and 1% Tween-20, pH 2.2) and re-incubated with rabbit anti-ERK1/2 antibody (1:1,000, Cell Signaling Technology, Cat. #9102, RRID:AB_330744) overnight, followed by visualization with Goat anti-rabbit-IgG DyLight 800 4× PEG-conjugated secondary antibody (1:15,000, Cell Signaling Technology, Cat. #5151, RRID:AB_10697505).

### Liquid chromatography-tandem mass spectrometry (LC-MS/MS)

Immunoprecipitated proteins were run on an 8% SDS-polyacrylamide gel, followed by staining in Coomassie gel staining buffer (1.5 mM Brilliant Blue R, 50% methanol, 10% glacial acetic acid). Bands of interest were excised for in-gel trypsin digestion. Briefly, proteins were reduced with 10 mM TCEP (Thermo Fisher Scientific) in 50 mM TEAB (Sigma-Aldrich), alkylated with 55 mM iodoacetamide in 50 mM TEAB (Sigma-Aldrich), followed by digestion with 5 μg/mL trypsin (Promega) in 25 mM TEAB (Sigma-Aldrich) for 16–18 h at 37 °C. The trypsinization was quenched with 0.1% trifluoroacetic acid (Sigma-Aldrich) in 50% acetonitrile (Merck). The resulting solution was evaporated with a Speed-Vac vacuum centrifugation system, topped up to 25–30uL with 0.1% formic acid (Merck), and analyzed by LC-MS/MS on either an LTQ Orbitrap Elite mass spectrometer (Thermo Fisher Scientific) or an Orbitrap Fusion Lumos mass spectrometer (Thermo Fisher Scientific) according to instrument availability. The LTQ Orbitrap Elite was the principal instrument used, with the Orbitrap Fusion Lumos was used to analyze samples obtained from 3 DGCR8 IPs of undifferentiated mESCs and 3 IP of undifferentiated FDC-P1 cells.

For LTQ Orbitrap Elite analyses, peptides were loaded onto a C18 trap column (75 μm × 2 cm, 100Å; Acclaim PepMap) and separated on a C18 analytical column (75 μm × 50 cm, 100Å; Acclaim PepMap). Peptides were eluted using a linear acetonitrile gradient containing 0.1% formic acid. Precursor ion mass spectra were acquired in positive ion mode over an m/z range of 375–1,650 at a resolution of 60,000. The 15 most intense precursor ions with charge state greater than or equal to two were selected for higher-energy collisional dissociation for the generation of data-dependent fragment ion mass spectra (MS/MS).

For Orbitrap Fusion Lumos analyses, peptides were loaded onto a Neo C18 trap cartridge (300 μm × 5 mm, 100Å; Acclaim PepMap) and separated on a C18 reversed phase column (75 μm × 25 cm, 120 Å; PepSep). Peptides were eluted using a linear acetonitrile gradient containing 0.1% formic acid. Precursor ion mass spectra were acquired in positive ion mode over an m/z range of 375–1,500 at a resolution of 120,000. The 20 most intense precursor ions with charge state greater than or equal to two were selected for higher-energy collisional dissociation for the generation of data-dependent fragment ion mass spectra (MS/MS).

For the analysis of DGCR8 in mESCs, eight independent purifications were performed on undifferentiated cells, and five on RA-differentiated cells. For the analysis of DGCR8 in FDC-P1 cells, 16 independent purifications were performed on undifferentiated cells, and five on RA-differentiated cells. For the analysis of DROSHA in mESCs, 11 independent purifications were performed on undifferentiated cells, and five on RA-differentiated cells.

### Mass spectrometric data processing and peptide quantification

Raw LC-MS/MS data were processed using Proteome Discoverer (v2.5; Thermo Fisher Scientific), and searches were performed using the Sequest HT algorithm within Proteome Discoverer against the *Mus musculus* UniProt/Swiss-Prot canonical database. Trypsin was taken as the proteolytic enzyme with full specificity, and up to two missed cleavages were allowed. Precursor and fragment mass tolerances were set to 10 ppm and 0.03 Da, respectively. Carbamidomethylation (C) was set as a fixed modification, while oxidation (M) and phosphorylation (S/T/Y) were included as dynamic modifications.

Peptide-spectrum matches were validated using Percolator based on q-values with a strict false discovery rate (FDR) of 1%. Peptide and protein identifications were validated at a 1% FDR using the Peptide Validator and the Protein FDR Validator, respectively. All proteins analyzed in this study were classified as high confidence based on the combined protein FDR assessment. Phosphorylation-site localization was assessed using IMP-ptmRS, with a site localization probability of greater than or equal to 75% considered acceptable for phospho-site assignment.

Peptide abundances were determined from the integrated MS1 precursor ion intensities in Proteome Discoverer. The phosphorylation frequency was estimated as the ratio of the abundance of the phosphorylated peptide to the total abundance of the corresponding peptide, regardless of its modification status.

### CRISPR/Cas9-mediated gene editing

Two guide RNA (gRNA) sequences were designed using TrueDesign Genome Editor (Invitrogen) and cloned into lentiCRISPRv2 (RRID:Addgene_52961) for each mutation (Sanjana et al., 2014). Single-stranded oligodeoxynucleotide (ssODN) templates were designed to incorporate the desired replacement sequence, flanked by 50-nt homology arms on both sides. The replacement sequence contained the target codon substitution together with additional silent mutations for PCR-based genotyping. A total of 5 µg lentiCRISPRv2_gRNA (2.5 µg each) and 10 µg ssODN were nucleofected into the MEF-depleted E14 mESC single cell suspension with the P3 Primary Cell 4D-Nucleofector® X Kit and 4D-Nucleofector® X Unit (Lonza), using the CG-104 program. Nucleofected cells were then plated onto mitomycin-inactivated puromycin-resistant MEFs. 24 h later, cells were selected with 2 μg/mL puromycin for 2 days 7 to 10 days after nucleofection, mESC colonies were individually isolated for expansion. The successful knock-ins were confirmed by PCR, followed by restriction enzyme digestion and Sanger sequencing of the corresponding regions. gRNAs and ssODN sequences used are provided in Supplementary Table S1, Supplementary Table S2, respectively. Primers for genotyping PCR are provided in Supplementary Table S3.

### Lentiviral CRISPR/Cas9-mediated DGCR8 knockout

MEF-depleted mESCs were transduced with lentiviruses generated from the lentiCRISPRv2 plasmid with or without DGCR8 exon 2-targeting gRNA (S238_gRNA_A, Supplementary Table S1). A plasmid without the guide RNA was employed as an empty vector (EV) control. Transduced cells were cultured in mESC medium supplemented with 3 µM CHIR99021 (Sigma-Aldrich) and 1 µM PD0325901 (Sigma-Aldrich) so that they could be maintained without inactivated MEFs. 24 h later, transduced cells were selected with 2 μg/mL puromycin for 2 days and then replated onto inactivated MEFs and cultured in mESC medium. 7 to 10 days later, mESC colonies were individually isolated for expansion. The successful knockout was confirmed by immunoblotting, followed by Sanger sequencing of the corresponding regions.

DGCR8 point mutants were generated by PCR and inserted into an MSCV-IRES-GFP retroviral vector. Single cell suspensions of DGCR8 deficient mESCs were then transduced with the retroviral supernatants by centrifuging flat bottom plates at 2000 rpm for 1.5 h at room temperature, followed by recovery at 37 °C, 10% CO_2_ for 1 h. The media were then replaced. Two days later, individual eGFP-positive cells were isolated by flow cytometric sorting. The resulting single-cell-derived clones were screened for eGFP expression and DGCR8 protein expression by immunoblotting. Clones expressing DGCR8 at level comparable to that in WT mESCs were selected for downstream analysis.

### Quantitative RT-PCR

Total RNA was extracted from cells with TRI Reagent (Sigma-Aldrich) and reverse transcribed using M-MuLV Reverse Transcriptase (New England Biolabs). Quantitative PCR was performed using GoTaq qPCR master mix (Promega) on a LightCycler 480 (Roche). Relative gene expression was calculated using the 2^−ΔΔCT^ method. Primers used are provided in Supplementary Table S4.

### *Chpf2* mRNA cleavage quantification

Poly(A)-containing RNAs were isolated from total RNA using a magnetic mRNA isolation kit (New England Biolabs, #S1550). A tag (5′–CAC​GAC​GCU​CUU​CCG​AUC​U) was then ligated to the isolated RNA with T4 RNA ligase, followed by reverse transcription with M-MuLV-RT (New England Biolabs). *Chpf2* cleavage product was pre-amplified with the primers 5′–CAC​GAC​GCT​CTT​CCG​ATC​T and 5′–GGT​GGA​GAG​GCT​AAG​TGC​TG for 20 cycles. Quantitative PCR was then performed on the pre-amplified using the primers 5′–CAC​GAC​GCT​CTT​CCG​ATC​T and 5′–TGT​TGG​CCT​GTT​CCT​GTT​CA. This was normalized to uncleaved *Chpf2* transcript levels detected with primers 5′–GCC​AAG​AAT​AGG​GAC​CCT​GAG​AAG and 5′–GGT​GTG​TTG​CTC​CGT​GAA​GTA​GT. *Actb* served as an additional control (Supplementary Table S4).

### Small RNA sequencing

Total RNA was extracted using TRI Reagent (Sigma-Aldrich) and treated with DNase I (New England Biolabs). Small RNA libraries were prepared with the Nextflex Small RNA v4 Library Prep with Blood miRNA Blockers kit (Revvity) and sequencing on a NovaSeq X in paired-end mode, with only Read one used for downstream analyses. Adapter sequences were removed using Trimmomatic. Trimmed reads were mapped to the mouse reference genome (GRCm38/mm10) using STAR (v2.7.11 b). MiRNA expression was quantified using featureCounts (Subread v2.1.1) with miRBase v22 annotations mapped to GRCm38. Multi-mapping reads were counted fractionally. Differential expression analysis was performed using the edgeR package. MiRNAs with |logFC| > 1 and a false discovery rate <0.05 were considered significantly differentially expressed.

### AlphaFold3 analysis

The Microprocessor complex was modelled using the AlphaFold server (https://alphafoldserver.com) (Abramson et al., 2024) with two molecules of M. musculus DGCR8 (UniProt ID: Q9EQM6), one molecule of M. musculus DROSHA (UniProt ID: Q5HZJ0), one heme molecule, two zinc ions, one calcium ion, and one pri-miR-16–2 (81-nt, extracted from PDB ID: 6V5B). Models were predicted either with or without specific phosphorylation on DROSHA or DGCR8. PDB templates were restricted to those available before 30/09/2021. The structural prediction data have been deposited in FigShare (doi: 10.26188/33435454). The predicted structures were visualized using PyMOL (version 3.1.7.2).

### Statistical analysis

Statistical analyses were performed using Prism 10 (GraphPad). Unpaired t-tests with Welch’s correction were used for comparisons between two groups. Multiple unpaired t-tests with Welch’s correction were used to compare the phosphorylation frequency of individual peptides between two groups. One-sample t-tests against a theoretical value of one were used to assess normalized ERK1/2 phosphorylation. Two-way ANOVA followed by uncorrected Fisher’s LSD test was applied for the qPCR experiments involving MEK1/2 inhibition. Two-way ANOVA with Tukey’s correction was applied for the qPCR experiments involving transduced mESCs and their differentiated derivatives. *P* < 0.05 was considered statistically significant.

## Results

### Purification of the endogenous microprocessor components

DROSHA and DGCR8, the main components of the Microprocessor complex, have been previously reported to be phosphorylated at multiple sites (Leong and Chong, 2025). Because most previous studies assessing the phosphorylation of the Microprocessor relied on overexpression of tagged protein, we first optimized purification of the endogenous DROSHA and DGCR8 proteins extracted from E14 mESCs via immunoprecipitation. Both DROSHA and DGCR8 could be extracted effectively with RIPA buffer; however, DGCR8 but not DROSHA could be immunoprecipitated with RIPA buffer effectively (Figure 1A). This suggests that RIPA buffer may be too strong to preserve efficient antibody-DROSHA interactions during DROSHA immunoprecipitation. Therefore, we tested different lysis buffer compositions as well as different primary antibody durations and temperatures to improve DROSHA immunoprecipitation efficiency (Supplementary Figures S1A,C). We found that weaker lysis buffers extracted DROSHA less efficiently and did not improve the final yield. While prolonged primary antibody incubation at 4 °C slightly improved the yield of DROSHA immunoprecipitation (Supplementary Figure S1C). Therefore, we used RIPA buffer to lyse cells for DROSHA immunoprecipitation, with prolonged primary antibody incubation at 4 °C.

**FIGURE 1:**
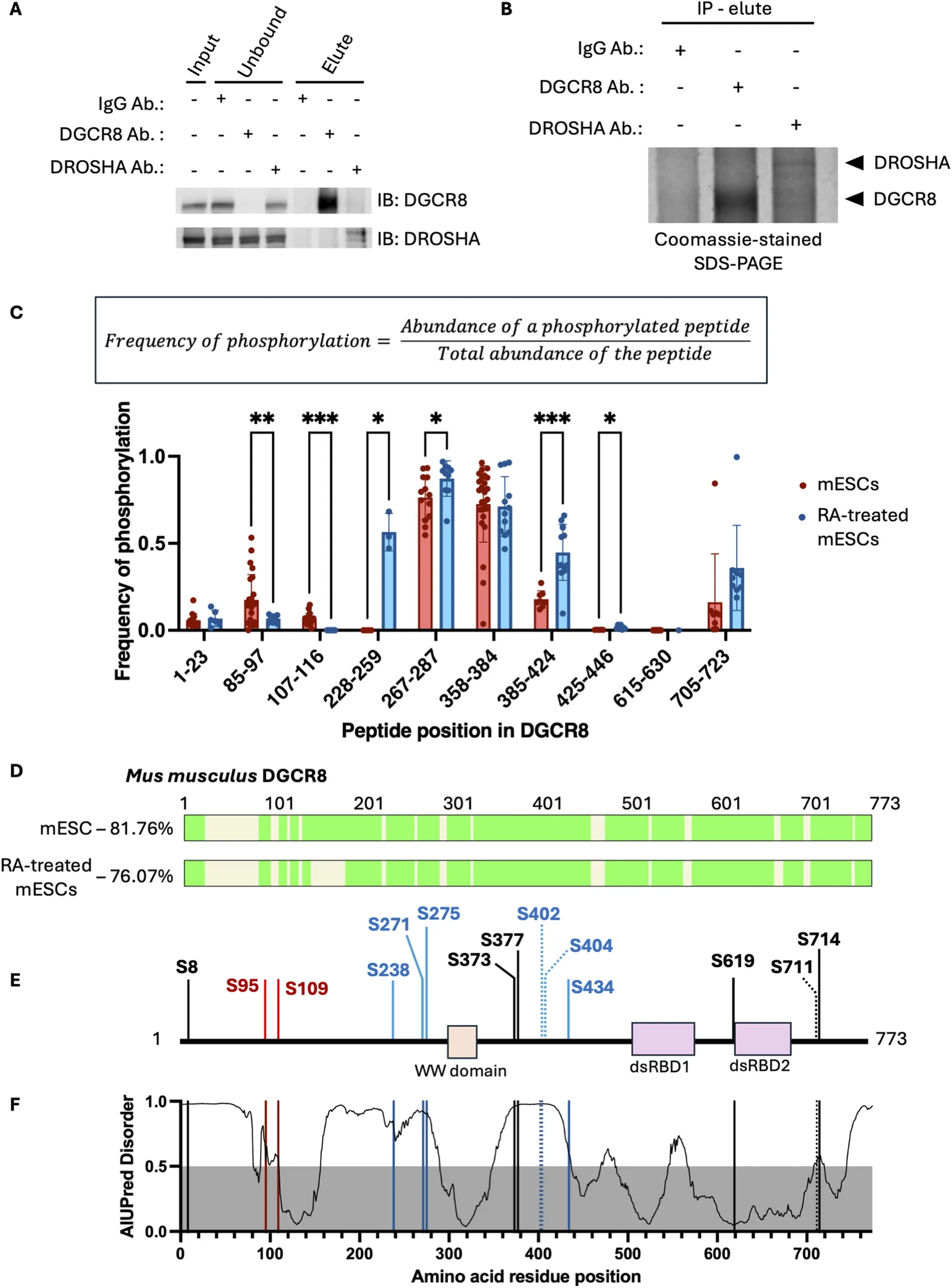
Dynamic phosphorylation of DGCR8 with differentiation. **(A)** Immunoblot confirming immunoprecipitation of endogenous DGCR8 and DROSHA from E14 mESCs. **(B)** Coomassie Blue-staining of the immunoprecipitated DGCR8 and DROSHA separated by SDS-PAGE. **(C)** Phosphorylation frequency of identified DGCR8 trypsin-digested peptides obtained from eight independent IPs of undifferentiated mESC cultures and five independent RA-differentiated cultures, from which 34 and 17 DGCR8 protein samples were analyzed, respectively. Error bars represent mean ± SD; *P < 0.05, **P < 0.01; ***P < 0.001; multiple unpaired t-test with Welch’s correction. **(D)** Mass spectrometric coverage of the immunoprecipitated DGCR8. **(E)** Summary of the phospho-sites in endogenous DGCR8 identified by mass spectrometry. **(F)** AIUPred disorder prediction of murine DGCR8. Solid lines indicate sites with 100% PTM site probability; dotted lines indicate sites with >99% and <100% PTM site probability; red indicates sites enhanced in mESCs; blue indicates sites enhanced in neural cells; black indicates non-differentially phosphorylated phospho-sites.

### DGCR8 is differentially phosphorylated upon neuronal differentiation

Next, we further purified the immunoprecipitated proteins via size separation through SDS-PAGE (Figure 1B). We excised bands of interest, trypsinized the proteins, and employed mass spectrometry to identify phosphorylated residues in trypsin-digested peptides. To determine which phosphorylation sites may be important for stem cell differentiation, we analyzed mESCs before and following *in vitro* differentiation into neuronal cells with retinoic acid (RA). We started with DGCR8 because of the superior purification achieved compared to DROSHA. We obtained overall DGCR8 coverages of 81.76% and 76.07% for DGCR8 extracted from mESCs and RA-treated mESCs, respectively (Figure 1D). Phosphorylation was identified by the presence of a characteristic mass shift of 79.966Da.

Since many of the trypsin-digested peptides contain more than one potential phosphorylation site, putative phosphorylation sites were grouped according to their corresponding trypsin-digested peptides. The frequency of phosphorylation for each peptide was estimated by dividing the abundance of the phospho-peptide by the total abundance of the corresponding peptide. By comparing the phosphorylation frequency estimates between the two cell differentiation states, two specific DGCR8 peptides appeared to have enhanced phosphorylation in mESCs and four other peptides to have enhanced phosphorylation in mESC-derived neuronal cells (Figure 1C). It should be emphasized that these phosphorylation frequency estimates are only semi-quantitative due to the label-free nature of the abundance quantification and do not present an absolute phosphorylation stoichiometry. However, this data does allow us to prioritize specific phosphor-sites for downstream functional analyses.

The localization of phospho-sites was assessed using the IMP-ptmRS algorithm within Proteome Discoverer, with a site probability threshold of 75%. The highest phospho-site localization possibilities for S8, S95, S109, S238, S271, S275, S373, S377, S434, S619, and S714 were reported as 100%. While the highest phospho-site localization possibilities for S402, S404, and S711 were 99.9%, 99.9%, and 99.4%, respectively (Supplementary Table S5). Supplementary Figure S2 presents the corresponding fragment ion mass spectra for their identification. Since S271 and S275, as well as S402 and S404, were identified together within the same phospho-peptides, the contribution of the individual phosphorylation sites to the differential phosphorylation frequency estimates could not be determined.

Consistent with the idea that phosphorylation preferentially occurs within intrinsically disordered protein regions, the prediction of the degree of disorder with AIUPred v2 (Erdős and Dosztányi, 2024) shows that most differentially phosphorylated and potentially differentially phosphorylated sites had an AIUPred disorder score greater than 0.5, suggesting that they are located within intrinsically disordered regions (Figure 1F; Supplementary Table S6). Furthermore, these sites are not located within any currently annotated DGCR8 domains (Figure 1E). The only exception was S619, which exhibited a very low phosphorylation frequency estimate value (0.07%) in neuronal cells.

We also analyzed the phosphorylation of endogenous DGCR8 in FDC-P1 cells, a mouse myeloid progenitor cell line, before and after differentiation into macrophages with GM-CSF. We obtained overall DGCR8 coverages of 78.01% and 68.82% for DGCR8 extracted from undifferentiated and differentiated FDC-P1 cells, respectively. Consistent with mESCs, the peptide spanning residues 267–287 of DGCR8 in FDC-P1 cells also appear to exhibit greater phosphorylation upon differentiation compared to the undifferentiated state (Supplementary Figure S3). Other phospho-peptides located at the N-terminus of DGCR8 exhibited a trend similar to that observed between differentiated and undifferentiated mESCs.

### Analysis of DROSHA phosphorylation

We also attempted to compare the DROSHA phosphorylation between differentiated and undifferentiated mESCs. Five phosphorylated regions in DROSHA, spanning residues 265–276, 339–359, 364–379, 801–808, and 1,095–1,105, were detected in mESCs, mESC-derived neuronal cells, or both (Supplementary Table S7). The phosphorylation of DROSHA at T272, S354, S356, S372, and S806 were supported by the corresponding fragment ion mass spectra, with the highest phospho-site localization possibilities of 100% (Supplementary Figure S4; Supplementary Table S7). However, the mass spectrometric data were not sufficient for phosphorylation frequency estimate comparisons due to the low yield obtained from DROSHA immunoprecipitation, which resulted in limited input for the mass spectrometry runs. This also reduced protein sequence coverage. We obtained overall DROSHA coverages of 55.79% and 48.43% for DROSHA extracted from mESCs and RA-treated mESCs, respectively. Although quantification was not possible, the data suggest that endogenous DROSHA, like DGCR8, may also undergo dynamic phosphorylation (Supplementary Table S7).

Together, these results suggest that phosphorylation of Microprocessor components, particularly DGCR8, changes during stem and progenitor cell differentiation.

### AlphaFold3 analysis of phosphorylation-associated microprocessor models

Most phospho-sites identified on DGCR8 and DROSHA are within predicted intrinsically disordered regions with poor structural information. Using AlphaFold3 to predict the conformation of the Microprocessor complex with the same component composition as the previously resolved cryo-EM structure (PDB ID: 6V5B) (Partin et al., 2020), most phospho-sites are found within regions with low model confidence scores (Supplementary Figure S5). The unmodified model had a predicted template modelling (pTM) score greater than 0.5 but an interface predicted template modelling (ipTM) score lower than 0.6, suggesting that while the overall fold may be broadly consistent with the true structure, the relative positioning of the subunits within the complex is less reliable (Zhang and Skolnick, 2004; Xu and Zhang, 2010). Models containing individual phosphorylation residues were different from the unmodified model in terms of conformation (Supplementary Figures S6, S7); however, these exploratory predictions require experimental validation.

### Generation of mESCs with specific phospho-deficient residues in DGCR8

To investigate whether differential DGCR8 phosphorylation might influence differentiation, we selected S95 and S238, representing phosphorylation sites enriched in undifferentiated mESCs and in neuronal cells differentiated from mESCs, respectively. The corresponding codons in the endogenous *Dgcr8* gene were mutated using the CRISPR/Cas9 system together with a single-stranded correction template. For each mutation, approximately 500 mESC colonies were picked and expanded for genotyping PCR. The successful knock-ins of the selected clones were further confirmed via PCR amplification of the genomic segment using primers outside the genomic segment corresponding to the correction template, followed by Sanger sequencing (Figure 2A). The S95A DGCR8 mESC clone was confirmed to be a homozygous mutant, and the S95A DGCR8 mESC clone was found to be a heterozygous S238A/- mutant, carrying S95A on one allele and disruption of the other allele. Both mutant mESC clones expressed DGCR8 protein at the same molecular weight as wild-type (WT) DGCR8 but with reduced abundance relative to WT (Figures 2B,C). The approximately 50% abundance in S238A/- mESCs is consistent with contributions from both WT alleles. Reduced DGCR8 abundance in S95A mESCs may reflect altered protein stability, as reported for DGCR8 phosphorylation; however, stability was not directly measured in this study.

**FIGURE 2:**
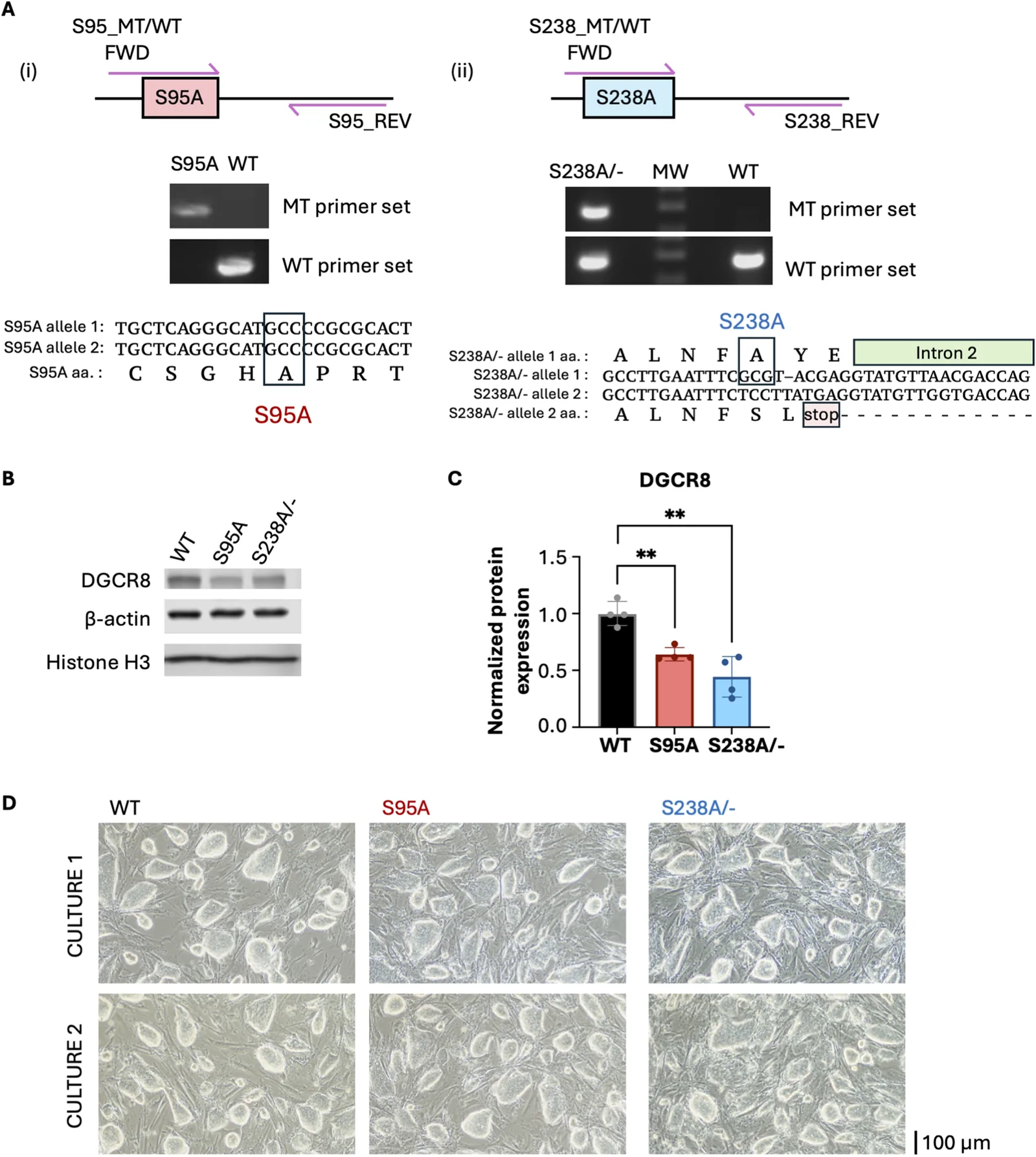
Characterization of S95A and S238A/- DGCR8 mESCs. **(A) **(I) S95A, and (ii) S238A/- DGCR8 phospho-mutant mESC clones generated by CRISPR-mediate gene editing. These were validated by PCR and Sanger sequencing. **(B)** Representative immunoblot for DGCR8 protein in the mutant mESCs. **(C)** Quantification of DGCR8 protein levels normalized to histone H3 over four experimental replicates. The mean ± SD is shown; **P < 0.01; unpaired t-test with Welch’s correction. **(D)** Representative bright field images of the mutant mESCs cultured on inactivated MEFs with LIF.

Both mutant mESCs formed colonies when grown on mouse embryonic fibroblasts (MEFs) that were indistinguishable from WT mESCs (Figure 2D). There was also no significant difference observed in the mRNA expression of the pluripotency markers, *Nanog* and *Oct4*, by both mutant mESCs (Figure 3A).

**FIGURE 3:**
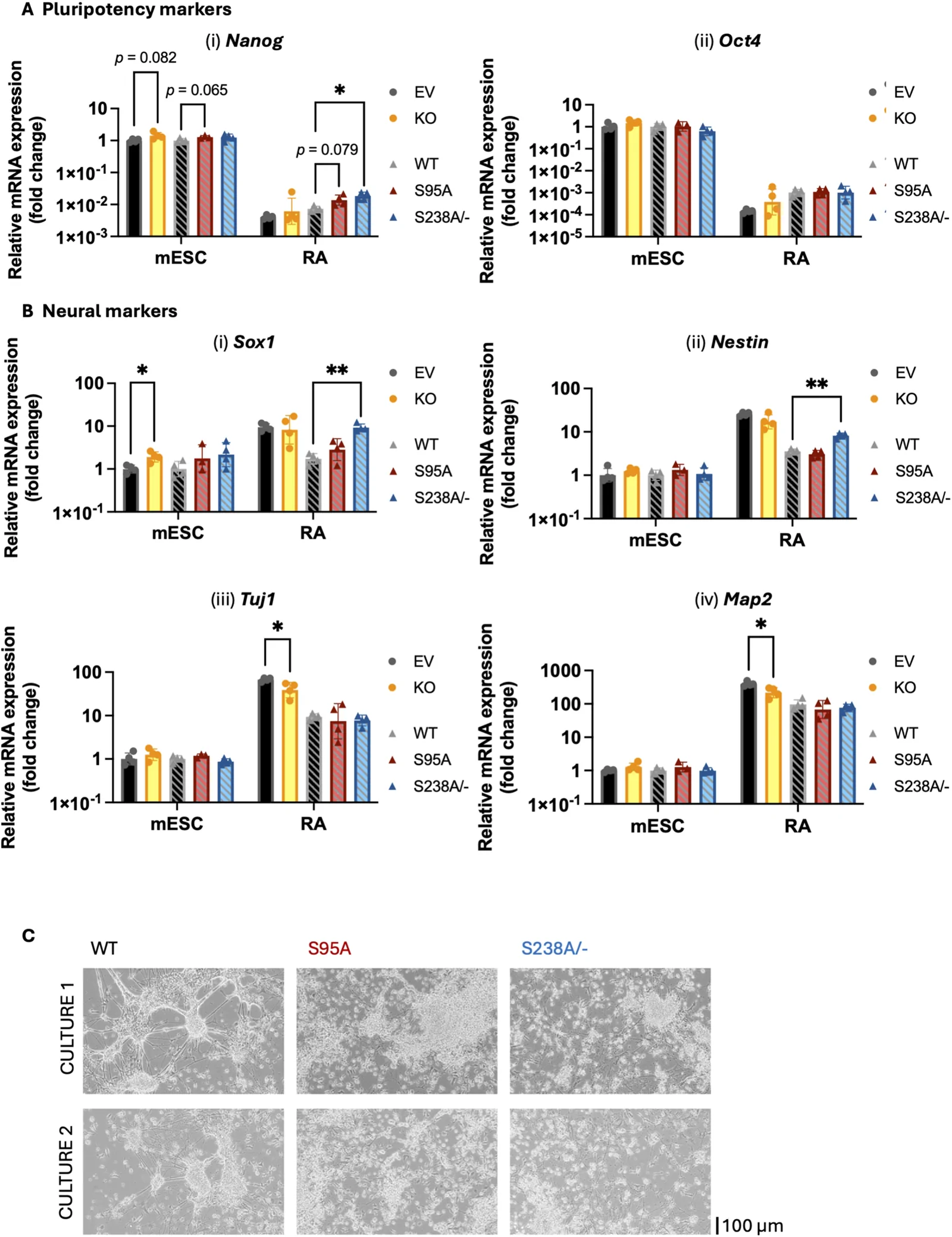
S95A and S238A/- DGCR8 mESCs exhibit impaired neuronal differentiation. mESCs were cultured in N2B27 medium with 1 µM RA for 2days, followed by a further 8 days without RA days. Quantitative RT-PCR analysis for expression of **(A)** pluripotency markers (i) *Nanog* and (ii) *Oct4* and **(B)** neuronal markers (i) *Sox1*, (ii) *Nestin*, (iii) *Tuj1*, and (iv) *Map2* in the mutant mESCs and following neuronal differentiation. For DGCR8 KO cells, mRNA expression levels were normalized to *Actb* mRNA and presented as relative to the mean expression of EV mESCs. For the endogenous mutant cells, mRNA expression levels were normalized to *Actb* and presented as relative to the mean expression of WT mESCs. Error bars represent geometric mean ± geometric SD; *P < 0.05; **P < 0.01; unpaired t-test with Welch’s correction. Only statistically significant differences are indicated. **(C)** Representative bright field images of neuronal cells differentiated from WT, S95A, and S238A/- DGCR8 mESCs.

### mESCs with S95 or S238 phospho-deficient DGCR8 exhibit altered differentiation potential

To determine whether the phenotypes observed in the phospho-deficient mutants could be explained by reduced DGCR8 expression, we assessed the differentiation potential of DGCR8 knockout (KO) mESCs. Wang, et al. previously generated a DGCR8-deficient allele by homologous recombination to excise exon 3, which generates a frameshift mutation (Wang et al., 2007). We generated a similar mutation by transducing WT mESCs with a plasmid encoding *Cas9* endonuclease and a guide RNA targeting the exon 2 – intron 2 junction. We then confirmed the generation of DGCR8 KO mESCs via Sanger sequencing of the corresponding genomic region. Both alleles of the *Dgcr8* gene in the DGCR8 KO mESC clone selected contained frameshift mutations (Supplementary Figure S8A). The loss of DGCR8 expression was confirmed by immunoblotting (Supplementary Figure S8B).

DGCR8 KO mESCs retained their ability to form mESC colonies (Supplementary Figure S8C). After 10 days of neuronal differentiation, the pluripotency markers *Nanog* and *Oct4* mRNA were significantly downregulated, and the mRNA expression of neuronal markers *Sox1*, *Nestin*, *Tuj1*, and *Map2* were significantly upregulated, indicating DGCR8 KO mESCs retained some ability to differentiate in response to RA (Figures 3A,B). However, the *Tuj1* and *Map2* mRNA expression levels were reduced in DGCR8 KO neuronal cultures compared with DGCR8 EV controls (Figure 3B), consistent with impaired neuronal differentiation.

When differentiated toward the neuronal lineage, both the S95A and S238A/- DGCR8 mESCs downregulated the pluripotency markers *Nanog* and *Oct4*, and the upregulated neuronal markers *Nestin*, *Tuj1*, and *Map2*, indicating that both DGCR8 phospho-deficient mutant mESCs retained the ability to undergo neuronal differentiation (Figures 3A,B). However, the pluripotency marker *Nanog* was not downregulated in the S238A/- mutant to the same extent as in WT or S95A cells with differentiation (Figure 3A). At the same time, the RA-differentiated S238A/- cells exhibited significantly higher levels of the neural progenitor markers *Sox1* and *Nestin*, compared to differentiated WT or S95A cells (Figure 3B). Interestingly, the S238A/- mutant expressed similar levels of the late neuronal markers *Tuj1* and *Map2* as WT and S95A cells by the end of the culture (Figure 3B). Neuronal network formation also appeared less extensive in the neuronal cultures of both mutants (Figure 3C). These observations are consistent with altered differentiation.

Next, we subjected the DGCR8 KO mESCs to spontaneous differentiation in embryoid body (EB) cultures. Like in the neuronal differentiation cultures, DGCR8 KO cells downregulated the mRNA expression of both pluripotency markers *Nanog* and *Oct4,* and upregulated the mRNA expression of three germ layer markers *Nestin*, *Gata6*, and *Hand1*, implying successful exit from pluripotency (Figures 4A–D). Compared with EV controls, DGCR8 KO EB cultures expressed higher levels of the pluripotency marker *Nanog* and significantly lower expression of *Tnnt2*, encoding a cardiomyocyte structural protein (Figures 4A,D). Consistent with the mRNA expression, beating cardiomyocytes were observed in DGCR8 EV but not in DGCR8 KO EB cultures by day 10 of spontaneous differentiation.

**FIGURE 4:**
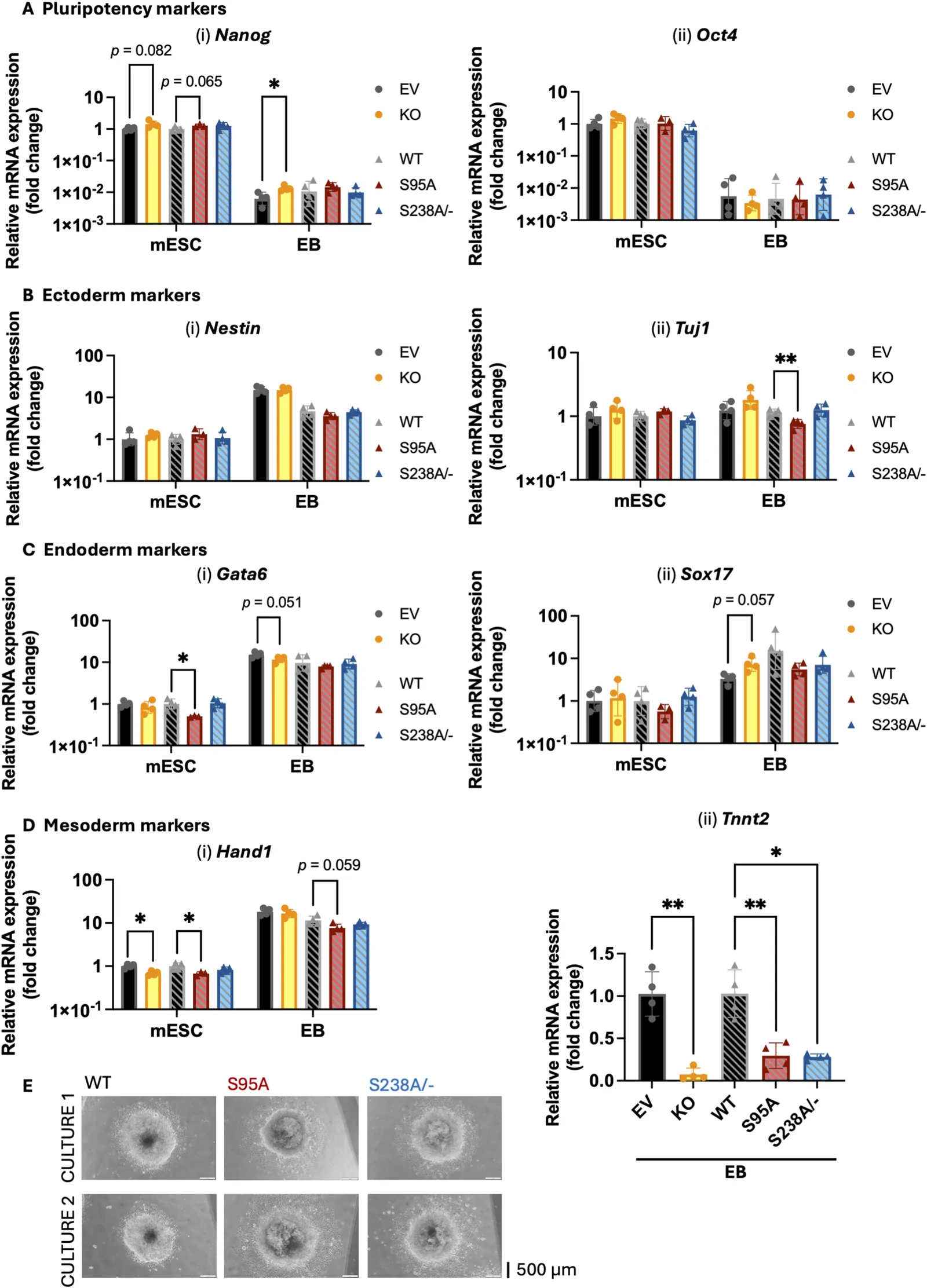
S95A and S238A/- DGCR8 mESCs exhibit impaired spontaneous differentiation in EB cultures. Embryoid bodies were generated from the mutant mESCs and seeded onto a 0.1% gelatin-coated plate in mESC medium without LIF for 10 days. Quantitative RT-PCR analysis for expression of **(A)** pluripotency markers (i) *Nanog* and (ii) *Oct4* **(B)** ectoderm markers (i) *Nestin* and (ii) *Tuj1* **(C)** endoderm markers (i) *Gata6* and (ii) *Sox17*, and **(D)** mesoderm markers (i) *Hand1* and (ii) *Tnnt2*. For DGCR8 KO cells, mRNA expression levels were normalized to *Actb* mRNA and presented as relative to the mean expression of EV mESCs (A-C,Di) or EV EB (Dii). For the endogenous mutant cells, mRNA expression levels were normalized to *Actb* mRNA and presented as relative to the mean expression of WT mESCs (A-C,Di) or WT EB (Dii). Basal *Nanog*, *Oct4*, *Nestin*, and *Tuj1* expression levels in mESCs are the same as those shown in Figures 3A,B. Error bars represent geometric mean ± geometric SD; *P < 0.05; **P < 0.01; unpaired t-test with Welch’s correction. Only statistically significant genotype comparisons are indicated. **(E)** Representative bright field images of WT, S95A, and S238A/- DGCR8 EBs on day 3 of cultures.

During spontaneous differentiation, both S95A DGCR8 and S238A/- DGCR8 mutants downregulated both *Nanog* and *Oct4* pluripotency markers to a similar extent as WT cells (Figure 4A). Although both mutants upregulated the markers of the three germ layers, the expression of the ectoderm maker *Tuj1* was significantly lower in S95A EB cultures than in WT EB cultures (Figures 4B–D). *Hand1* expression also showed a trend of reduced expression in S95A EB cultures (Figure 4D). Furthermore, the expression level of *Tnnt2* was significantly lower in both S95A and S238A/- DGCR8 EB cultures than in WT EB cultures (Figure 4D). These findings imply that both mutations are impairing the spontaneous differentiation of mESCs, but to different degrees. Consistent with this, beating cardiomyocytes were observed in WT EB culture as early as Day 7, while no beating cardiomyocytes were observed in either S95A or S238A/- EB cultures by Day 10 of spontaneous differentiation (Supplementary Video S1). Moreover, cavitation occurred earlier in the mutant EBs than in the WT EBs (Figure 4E).

### Re-expression of S95A and S238A DGCR8 mutants in DGCR8 deficient cells exhibit enhanced *nanog* mRNA expression across differentiation conditions

To further investigate if the two phospho-site in DGCR8 influences the stem cell differentiation outcomes, we next introduced DGCR8 phospho-deficient mutants into the DGCR8 KO mESCs, by transducing with retroviral constructs to re-express WT, S95A, S109A, or S238A DGCR8. For each construct, two clones with comparable DGCR8 expression levels to untransduced WT mESCs were selected for downstream analysis (Supplementary Figure S9).

All mESC clones exhibited downregulated pluripotency markers, *Nanog* and *Oct4*, after neuronal or spontaneous differentiation (Supplementary Figure S10). No significant difference was observed among the mutants within the undifferentiated mESCs, the neuronal cultures, or the EB cultures. However, both S95A and S238A DGCR8-expressing cells showed higher *Nanog* mRNA expression than WT DGCR8-expressing cells following RA or EB differentiation.

Collectively, these results indicate that the S95A and S238A/- DGCR8 clones exhibited altered mESC differentiation outcomes.

### S95A and S238A/- DGCR8 mESCs exhibit mild alterations in mature miRNA expression profiles

To investigate whether the phospho-deficient mutations impair miRNA processing activity of the Microprocessor complex, small RNA sequencing was performed to examine the miRNA profiles of WT, S95A, and S238A/- DGCR8 mESCs. Only six miRNAs were upregulated, and 13 were downregulated in the S95A mutant (Figure 5A; Supplementary Table S8), while 25 miRNAs were upregulated and eight were downregulated in S238A/- DGCR8 mESCs compared with WT mESCs (Figure 5B; Supplementary Table S9). Since phosphorylation at S238 was not detected in undifferentiated mESCs, the altered miRNA profile observed in S238A/- DGCR8 mESCs may reflect reduced DGCR8 protein expression rather than loss of S238 phosphorylation. Although S95A DGCR8 mESCs were also shown to express less DGCR8 protein, most of the differentially expressed miRNAs were not shared between S95A DGCR8 and S238A/- DGCR8 mutants (Figure 5C). This suggests that the altered miRNA profile in S95A DGCR8 mESCs is unlikely to be explained solely by reduced DGCR8 expression and may instead result from the loss of S95 phosphorylation.

**FIGURE 5:**
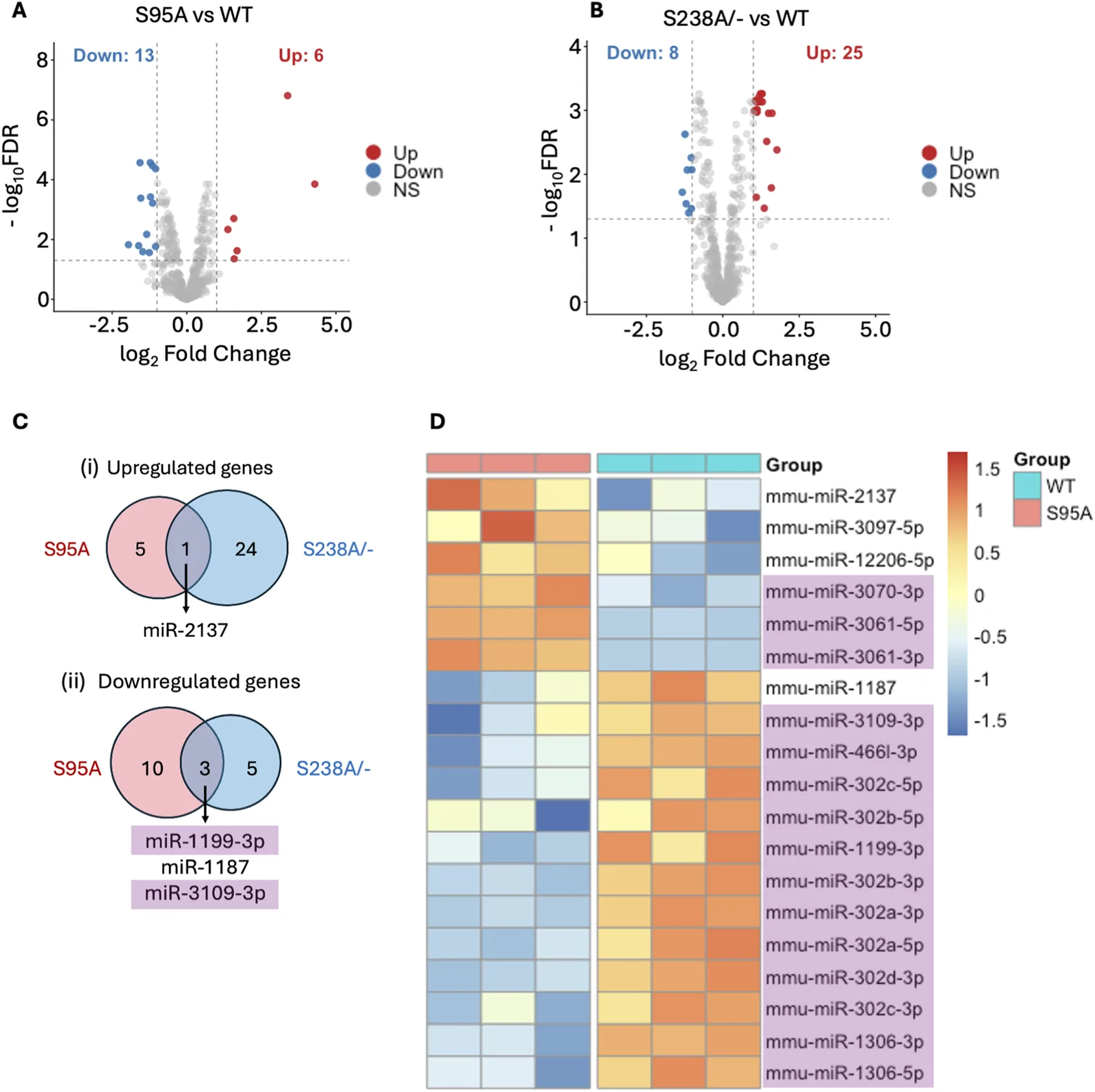
S95A DGCR8 mESCs exhibit a mildly altered miRNA profile. Volcano plot of differentially expressed miRNAs in **(A)** S95A DGCR8 mESCs and **(B)** S238A/- DGCR8 mESCs. **(C)** Shared (i) upregulated miRNAs and (ii) downregulated miRNAs between S95A DGCR8 and S238A/- DGCR8 mESCs. **(D)** Heatmap of the differentially expressed miRNAs in S95A DGCR8 mESCs compared to WT mESCs. MiRNAs with high miRBase annotation confidence are highlighted in purple.

The miRNAs specifically upregulated in S95A DGCR8 mESCs (Figure 5D; Supplementary Table S8) include those with low miRBase annotation confidence (miR-3097 and miR12206) or unknown functions (miR-3070 and miR-3061) (Kozomara et al., 2019). Interestingly, *pri-miR-3070* has a large internal loop, while *pri-miR-3061* contains an almost-perfect stem, which differ considerably from typical canonical miRNA stem-loop structures (Supplementary Figures S11A–C). On the other hand, miR-1306, miR-466L, miR-302a, miR-302b, miR-302c, and miR-302 d are the downregulated miRNAs in S95A DGCR8 mESCs (Figure 5D; Supplementary Table S8).

### S95A mESCs, but not S238A/- mESCs, exhibit enhanced *Chpf2* mRNA cleavage

In addition to processing pri-miRNA transcripts, the Microprocessor complex also recognizes mRNAs containing hairpins resembling those of the pri-miRNAs (Lee and Shin, 2017). To investigate whether the phospho-deficient mutations alter the mRNA cleavage activity of the Microprocessor complex, we extracted total RNA from mESCs and further isolated poly(A)-containing RNAs to capture all uncleaved mRNAs and any cleaved 3′ mRNA remnants. Endonucleolytic cleavage mediated by DROSHA generates a phosphate group at the 5′ end of the 3′ RNA cleavage product (Court et al., 2013). The presence of this 5′ phosphate group allowed selective ligation of an RNA linker to the 5′ end of the cleaved RNAs in the isolated RNAs. Following reverse transcription and PCR amplification using a primer targeting the 5′ linker and a gene-specific primer, we were able to detect Microprocessor-generated mRNA cleavage products.

From our previous degradome sequencing data, a degradome product mapping to *Chpf2* mRNA exon 4 was found to be significantly downregulated (0.14-fold relative to WT, FDR = 0.0073, P-value = 6.6 × 10^−7^) in DROSHA-deficient mESCs, suggesting that *Chpf2* is a Microprocessor mRNA cleavage target (Supplementary Figure S12A) (Gu et al., 2024). The 5′ end of the piled-up reads represents the cleavage site, and the region preceding it was predicted to fold into a stem-loop structure (Supplementary Figure S12B). By quantitative PCR, the abundance of cleaved *Chpf2* mRNA relative to uncleaved *Chpf2* mRNA was shown to be significantly increased in S95A DGCR8 mESCs compared with WT mESCs, whereas no significant differences were observed in the uncleaved *Chpf2* mRNA levels expressed in WT, S95A, S238A/- DGCR8 mESCs (Figure 6A; Supplementary Figure S12C), suggesting that S95 phosphorylation influences cleavage of this mRNA target.

**FIGURE 6:**
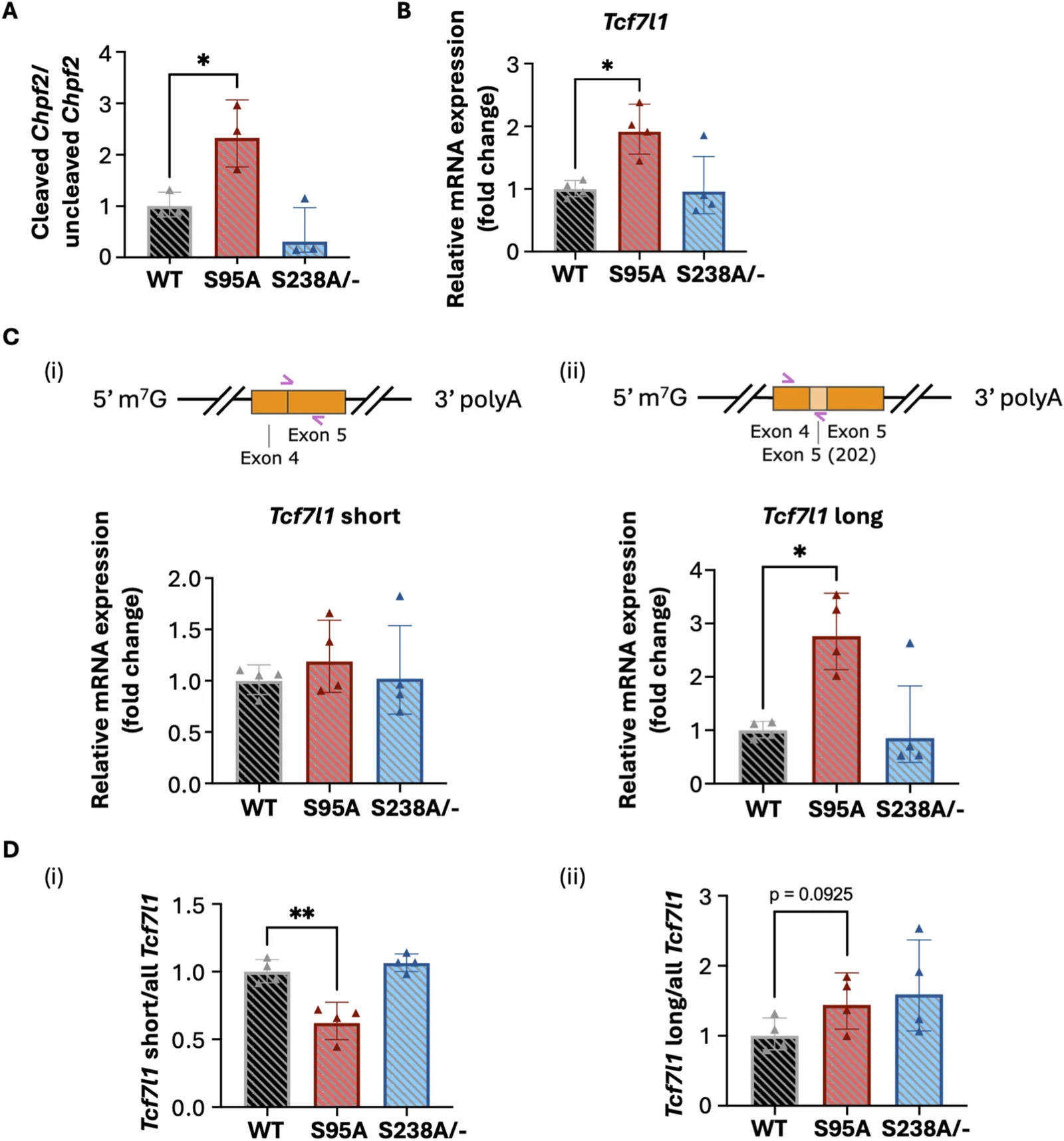
The S95A DGCR8 mutation enhances *Chpf2* mRNA cleavage and alters *Tcf7l1* isoform composition in mESCs. **(A)** Quantitative RT-PCR analysis for the cleaved *Chpf2* 3′ remnant in DGCR8 mutant mESCs. The cleaved mRNA levels were normalized to uncleaved *Chpf2* mRNA. **(B)** Quantitative RT-PCR analysis for total *Tcf7l1* mRNA. The mRNA expression levels are normalized to *Actb* mRNA. **(C)** Quantitative RT-PCR analysis for (i) *Tcf7l1* short and (ii) *Tcf7l1* long isoform mRNAs normalized to *Actb.* **(D)** Quantitative RT-PCR analysis for (i) *Tcf7l1* short and (ii) long isoforms normalized to total *Tcf7l1* mRNA. All the mRNA expression levels were normalized relative to the mean expression of WT mESCs. Error bars represent geometric mean ± geometric SD; *P < 0.05; **P < 0.01; unpaired t-test with Welch’s correction.

### S95A but not S238A/- mESCs exhibited altered *Tcf7l1* mRNA isoform composition

Recognition of an mRNA-embedded hairpin by the Microprocessor can also regulate alternative splicing of the target mRNA. This was shown for the *Tcf7l1* mRNA, encoding a regulator of early embryonic development. A DGCR8 mutant with disruption of all 23 potential phosphorylation sites was previously shown to result in increased splicing of the long isoform of *Tcf7l1* mRNA at the expense of the short isoform (Cirera-Salinas et al., 2017).

S95 was one of the 23 phosphorylation sites that were mutated in that study. Consistent with a requirement of DGCR8, expression of the *Tcf7l1* long isoform was increased in DGCR8 KO mESCs (Supplementary Figures S13B,C). Total *Tcf7l1* mRNA levels were significantly upregulated in S95A DGCR8 mESCs (Figure 6B). Specifically, it was the long isoform that was upregulated in S95A DGCR8 mESCs, altering the isoform composition (Figures 6C,D). Consistent with the idea that S238 is not phosphorylated in mESCs, no significant difference was observed in S238A/- DGCR8 mESCs.

### Transcription of poised genes is unaffected in S95A and S238A/- mESCs

DGCR8 has also been reported to sequester the transcription co-activator FLII for the transcription inhibition of poised genes (Lyu et al., 2024). However, we did not observe any changes in the mRNA expression of *Chic1 or Rev3l* in either mutant, which are two of the reported poised genes regulated by both DGCR8 and FLII (Supplementary Figures S14A,B) (Lyu et al., 2024). Thus, phosphorylation at neither S95 nor S238 appears necessary for sequestering FLII.

### ERK/MAPK signaling may regulate DGCR8 S95 phosphorylation

A MEK1/2 inhibitor was reported to impair phosphorylation of DGCR8 at one or more of 23 candidate phosphorylation sites, suggesting that DGCR8 phosphorylation may be regulated by the ERK/MAPK signaling pathway (Herbert et al., 2013; Zhu et al., 2015). ERK1 and ERK2 are the downstream kinases of MEK1/2 (Eblen, 2018). ERK1 was predicted by PhosphoSitePlus to be a potential kinase for DGCR8 S95 phosphorylation, with a site percentile score of 90.44 (Supplementary Table S10) (Hornbeck et al., 2015; Johnson et al., 2023).

To assess if reduced ERK1/2 activity has the same impact as the S95A mutation, we treated WT mESCs with PD0325901, a highly selective MEK1/2 inhibitor (Barrett et al., 2008). Reduced ERK1 and ERK2 phosphorylation was observed, confirming MEK1/2 inhibition (Figures 7A–C; Supplementary Figures S14A,B). MEK1/2 inhibition led to a mild but non-significant increase in *Nanog* mRNA expression and a significant reduction in *Gata6* mRNA expression, which was comparable to the DMSO-treated S95A DGCR8 mESCs (Figures 7D,E). MEK1/2 inhibition also downregulated *Hand1* mRNA expression in WT mESCs, partially resembling the effect observed in S95A DGCR8 mESCs (Figure 7F). However, MEK1/2 inhibition exhibited a stronger effect than the S95A mutation on the expression of these differentiation-associated markers, and the effects of MEK1/2 inhibition on *Nanog* and *Hand1* mRNA expression were retained in DGCR8 KO mESCs (Figures 7D–F; Supplementary Figures S15C).

**FIGURE 7:**
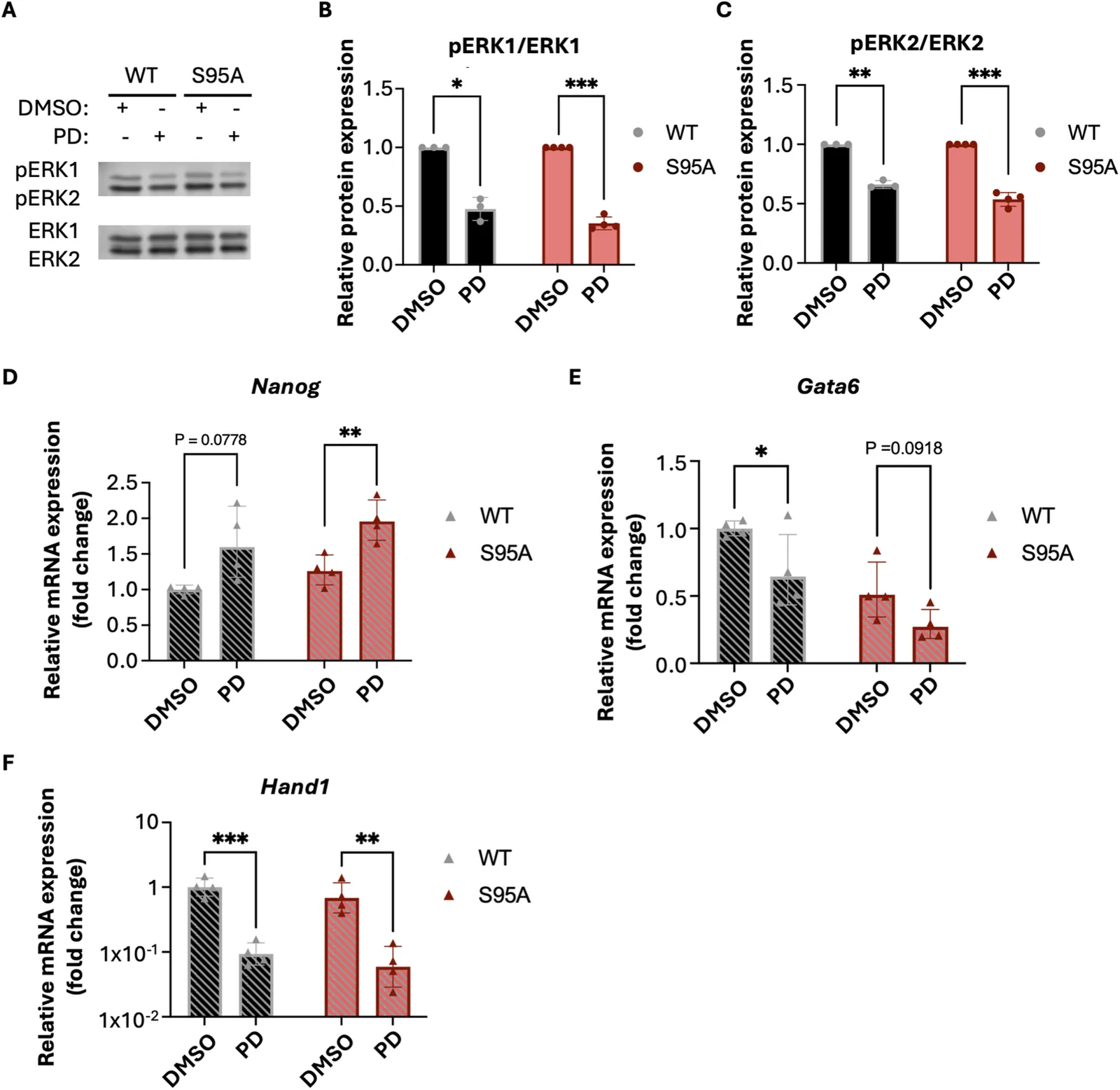
MEK1/2 inhibition in mESCs partially phenocopies the S95A DGCR8 mutant. **(A)** Representative immunoblot for ERK1/2 and pERK1/2 protein levels in WT and S95A DGCR8 mESCs treated with either 0.01% DMSO or 1 μM PD0325901 (PD) for 24 h. Quantification of **(B)** pERK1 and **(C)** pERK2 protein levels, normalized to ERK1 and ERK2, respectively, from four independent cultures. pERK1 and pERK2 expression levels are relative to the corresponding DMSO-treated cultures. Error bars represent mean ± SD; *P < 0.05; **P < 0.01; ***P < 0.001; one-sample t-test against a relative value of 1. Quantitative RT-PCR analysis of **(D)** *Nanog* **(E)** *Gata6*, and **(F)** *Hand1* mRNA expression levels in mESCs treated with either 0.01% DMSO or 1 μM PD0325901 for 24 h. mRNA expression levels were normalized to *Actb* mRNA and are presented relative to the mean expression of WT mESCs treated with DMSO. Error bars represent geometric mean ± geometric SD; *P < 0.05; **P < 0.01; ***P < 0.001; two-way ANOVA with uncorrected Fisher’s LSD test. Only statistically significant comparisons are indicated.

Together, these findings are consistent with the possibility that ERK/MAPK signaling might contribute to the regulation of DGCR8 S95 phosphorylation, but further validation is required to establish S95 as a direct ERK1/2 substrate. The broader effects of MEK1/2 inhibition and the persistence of some transcriptional responses in DGCR8 KO mESCs suggest that either ERK/MAPK signaling has additional roles in maintaining stem cell pluripotency and facilitating differentiation beyond DGCR8 S95 phosphorylation or that there are additional non-specific effects of the MEK1/2 inhibitor.

Altogether, the presented results support the concept that phosphorylation of specific residues in DGCR8 regulates Microprocessor-dependent processes.

## Discussion

Although phosphorylation of DROSHA and DGCR8 has been reported previously, many of these phosphorylation sites were identified via overexpression of tagged proteins. In our study, we mapped phosphorylation sites of the endogenous proteins in mESCs before and after differentiation *in vitro*, providing evidence that these modifications do indeed occur at endogenous protein levels without ectopic overexpression, and that these phosphorylations are dynamic and can alter with cell differentiation states. Moreover, through structural prediction, we raised the possibility that phosphorylation of the Microprocessor components affects the complex conformation.

In our study, phosphorylation at S238 was not detectable in mESCs and is only phosphorylated upon differentiation. Consistent with this, the disruption of S238 phosphorylation did not show any significant changes in pluripotency and lineage-specific markers, as well as the noncanonical Microprocessor activities. Nevertheless, the S238A/- DGCR8 mutant exhibited altered differentiation phenotypes, particularly during neuronal differentiation, where the mRNA expression of neural progenitor transcription factor *Sox1* and neural progenitor intermediate filament protein *Nestin* remained elevated.

However, the interpretation of the observations in the S238A/- mutant is complicated due to the heterozygous knockout genotype. Although the S238A/- DGCR8 mESC clone expressed less DGCR8, previous studies suggest that DGCR8 level is not limiting in pluripotent stem cells or during early lineage development. Complete knockout of DGCR8 is embryonic lethal in mice (Wang et al., 2007), while heterozygous DGCR8 knockout mice can be born, have a normal life expectancy, and are fertile (Ouchi et al., 2013). Similarly, heterozygous DGCR8 knockout human ESCs maintained stem cell pluripotency and could differentiate into all three germ layer lineages (Reé et al., 2022). In addition, DGCR8 heterozygous knockout mESCs exhibit normal miRNA profiles (Wang et al., 2007), suggesting a moderate reduction in DGCR8 expression is tolerated and is unlikely to produce a phenotype equivalent to complete DGCR8 loss. However, heterozygous DGCR8 deficiency has been associated with altered miRNA profile and reduced neural progenitor cells in adult mice (Ouchi et al., 2013), suggesting that dosage could be important in specific cellular or developmental contexts. Therefore, even though S238A/- cells did not fully phenocopy DGCR8 KO cells, the reduced DGCR8 dosage may contribute to the observed S238A/- phenotypes.

On the other hand, the mutation of S95 altered both neuronal and spontaneous differentiation outcomes. It exhibited a modest, non-significant increase in the expression of pluripotency marker *Nanog* and a significant reduction in the expression of lineage-specifying transcription factors *Gata6* and *Hand1* in mESCs. After spontaneous differentiation, the absence of beating cardiomyocytes and the reduced mRNA expression of lineage-specific structural proteins *Tuj1* and *Tnnt2* in the S95A EB cultures indicate altered differentiation outcomes.

We must acknowledge an important limitation of this study. Despite extensive screening, we recovered only one correctly targeted clone per mutation in this study. Thus, clonal artefacts remain possible. However, the results from the re-expression of DGCR8 phospho-deficient mutants in DGCR8 KO mESCs were consistent with the CRISPR-mutant phenotype.

Small RNA sequencing revealed an altered miRNA expression profile in S95A DGCR8 mESCs. Although the number of differentially expressed miRNAs was relatively small, this is consistent with the idea that phosphorylation fine-tunes Microprocessor activity rather than globally controlling miRNA processing (Herbert et al., 2013). In terms of the upregulated miRNAs with low miRBase annotation confidence, the miR-3097 hairpin spans the intron three – exon 4 junction of *Rgs12,* while the miR-12206 hairpin is located within the intron one of *Ccdc71*. The processing of these miRNAs may in fact regulate the mRNA expression of the host genes instead. The other upregulated miRNAs with high miRBase annotation confidence but unknown function, miR-3070 and miR-3061, have atypical stem-loop structures in their nascent transcripts. These observations suggest that the phosphorylation of S95 may influence DGCR8 substrate selectivity.

Of the differentially downregulated miRNAs, miR-1306 is derived from the second stem-loop embedded within the 5′UTR of *Dgcr8* mRNA (Han et al., 2009). Because this hairpin is located only 80bp from the edited S95A genomic region, the observed reduction in miR-1306 expression could potentially be influenced by the local sequence context of the *Dgcr8* transcript rather than because of altered DGCR8 protein function *per se*.

Of the other miRNAs, miR-302a, miR-302b, miR-302c, and miR-302 d originate from a single polycistronic miRNA cluster that is enriched in ESCs (Suh et al., 2004). Given that the expression of the stem cell pluripotency markers *Nanog* and *Oct4* were unchanged, the downregulation of miR-302a, miR-302b, miR-302c, and miR-302 d levels are unlikely to be secondary to a global loss of pluripotency. Instead, it may indicate altered processing efficiency of specific DGCR8-dependent hairpins.

The Microprocessor cleaves stem-loop containing mRNAs in stem and progenitor cells, and the cleavage can either promote or inhibit differentiation (Johanson et al., 2015; Knuckles et al., 2012; Rolando et al., 2016). We showed that disruption of S95 phosphorylation increased the ratio of cleaved *Chpf2* transcript, suggesting that phosphorylation can modulate Microprocessor-mediated mRNA cleavage. Moreover, the S95A mutation significantly increased the expression of *Tcf7l1* long isoform, resulting in altered isoform composition. Increased expression of the *Tcf7l1* long isoform has been reported to be associated with maintenance of stem cell pluripotency and inhibition of lineage-associated gene expressions (Cirera-Salinas et al., 2017; Wray et al., 2011; Yi et al., 2011). Mutation of multiple DGCR8 phosphorylation sites has been shown to similarly promote the expression of the *Tcf7l1* long isoform and impair exit from pluripotency (Cirera-Salinas et al., 2017). Since S95 is among the phosphorylation sites mutated in that study, our findings suggest that S95 is an important contributor to the regulation of DGCR8-dependent alternative splicing. Collectively, these findings suggest that S95 phosphorylation contributes to the maintenance of mESC differentiation potential, potentially via regulation of canonical miRNA biogenesis, *Tcf7l1* alternative splicing, and Microprocessor-mediated mRNA cleavage.

The ERK/MAPK pathway has been reported to facilitate both the maintenance of pluripotency and differentiation (Duan et al., 2026; 2025; Kim et al., 2012; 2014; Kunath et al., 2007; Tsanov et al., 2017; Ying et al., 2008; Yu et al., 2024). For example, activation of ERK1/2 has been reported to drive mouse embryonic stem cells to exit from pluripotency, whereas simultaneous inhibition of MEK1/2 and GSK3 drives mESCs to the ground state (Kunath et al., 2007; Ying et al., 2008). Consistent with previous reports linking DGCR8 phosphorylation to ERK/MAPK signaling, S95A DGCR8 mESCs had reduced DGCR8 protein expression, and MEK1/2 inhibition partially phenocopied the transcriptional effects of the S95A mutation in mESCs. ERK1/2 have been reported to phosphorylate and regulate the stability and activity of pluripotency-associated factors KLF4, NANOG, LIN28, and ESRRB (Duan et al., 2025; Kim et al., 2012; 2014; Tsanov et al., 2017), as well as RNA regulatory proteins DCP1A and HNRNPF (Duan et al., 2026; Yu et al., 2024), all of which also contribute to the regulation of mESC pluripotency and differentiation. Our findings suggest a possible association between ERK/MAPK signaling and DGCR8 S95 phosphorylation; however, further investigation is required to determine whether ERK1/2 directly phosphorylates S95.

In summary, our study supports the idea that DGCR8 phosphorylation serves as an important regulatory mechanism controlling Microprocessor activity and stem cell differentiation outcomes.

## Data Availability

The datasets presented in this study can be found in online repositories. The names of the repository/repositories and accession number(s) can be found below: https://www.ncbi.nlm.nih.gov/, GSE336265.
