# Supplementary material for: Dynamic phosphorylation of DGCR8 regulates embryonic stem cell differentiation and microprocessor function
Source: Front Cell Dev Biol. 2026 Oct 1;14:1918038. doi: 10.3389/fcell.2026.1918038 (PMC13669187; doi:10.3389/fcell.2026.1918038)
Supplement: Supplementary file 1 [file Supplementaryfile1.pdf]

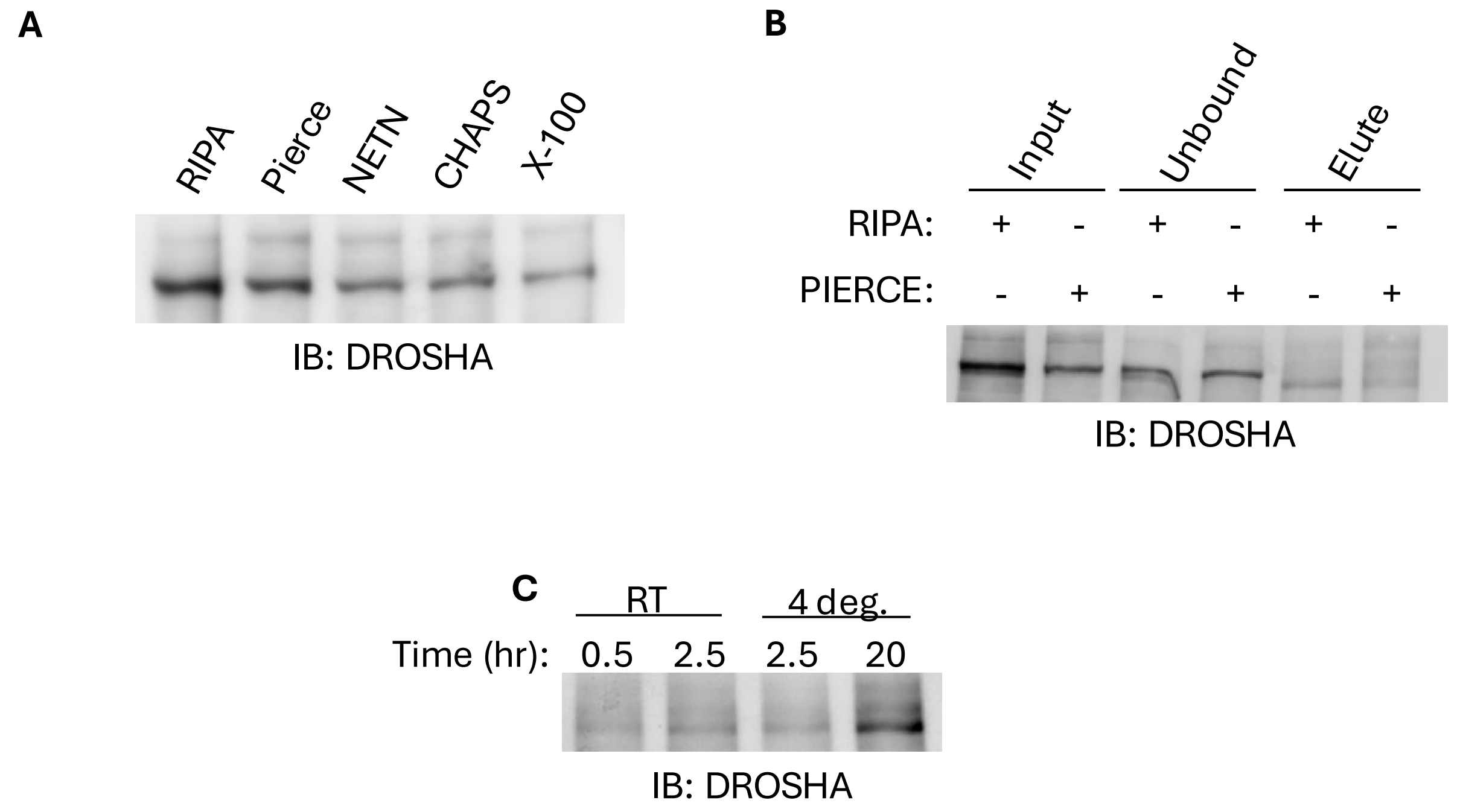

Supplementary Figure 1. Optimizing the immunoprecipitation of endogenous DROSHA from mESCs. (A) Immunoblot for endogenous DROSHA extracted from same number of mESCs lysed with RIPA, Pierce (25 mM Tris-HCl pH 7.4, 150 mM NaCl, 1 mM EDTA, 1% NP-40 and 5% glycerol), NETN (20 mM Tris-HCl, pH 8.0, 100 mM NaCl, 1 mM EDTA, 0.5% NP-40), CHAPS (25 mM Tris-HCl, pH 7.5, 120 mM NaCl, 1 mM EDTA, 0.33% CHAPS) and X-100 (20 mM Tris-HCl, pH 8.0, 137 mM NaCl, 2 mM EDTA, 1% Trition X-100) lysis buffers. **(B)** Immunoblot for DROSHA immunoprecipitated using the RIPA or Pierce lysis buffers. **(C)** Immunoblot for immunoprecipitated DROSHA in RIPA lysis buffer, with 0.5 or 2.5 hrs primary antibody incubation at room temperature (RT) or 2.5 or 20 hrs at 4°C. 1/10 of the elutes were loaded.

pSer8

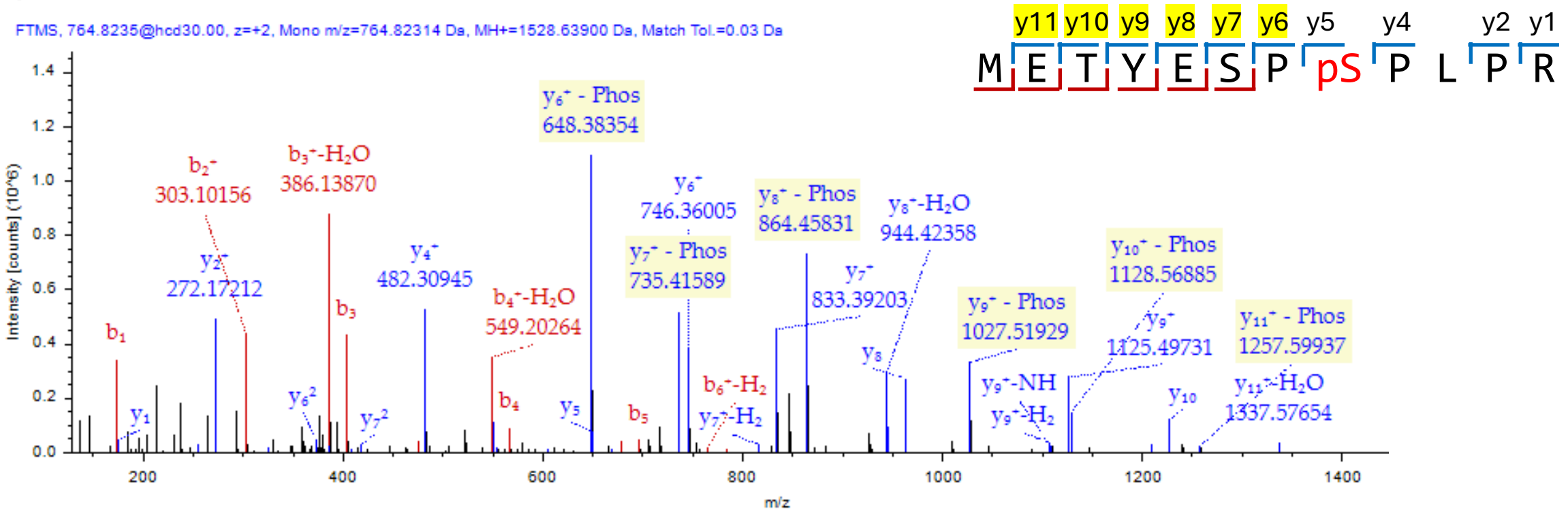

pSer95

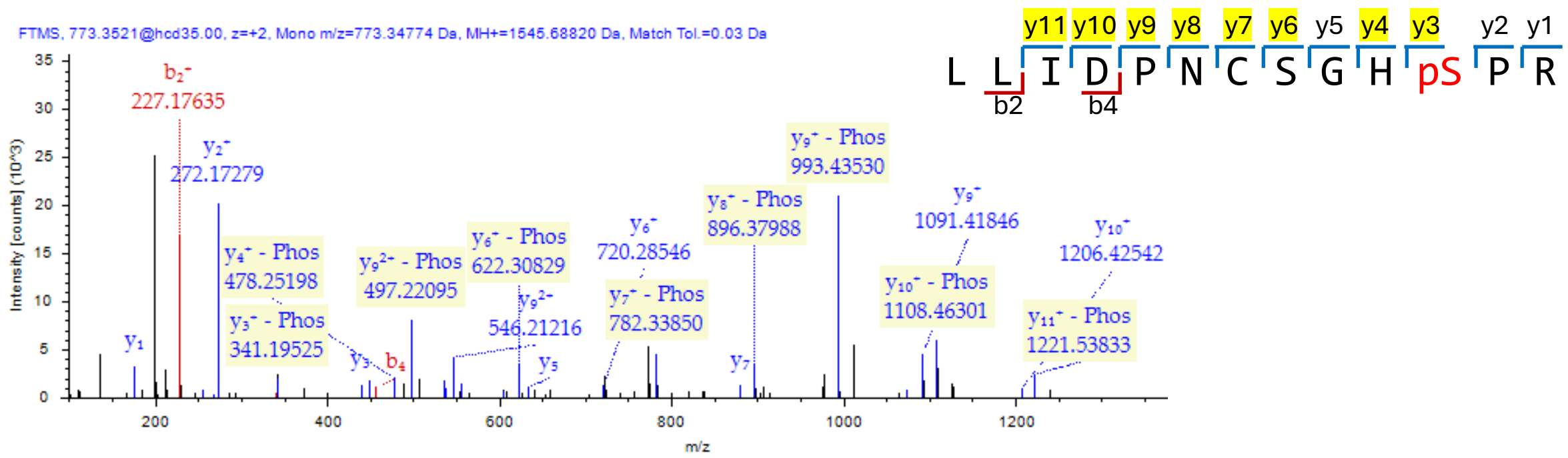

pSer109

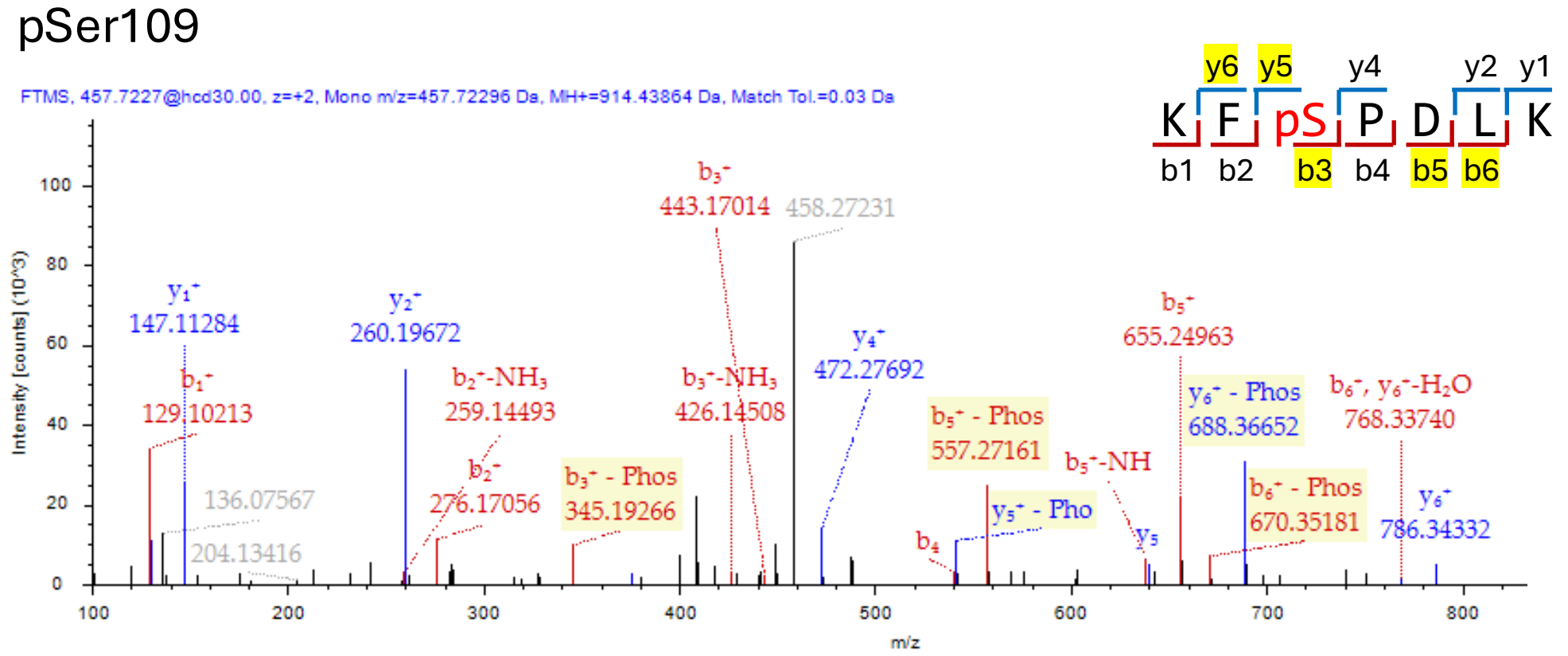

pSer238

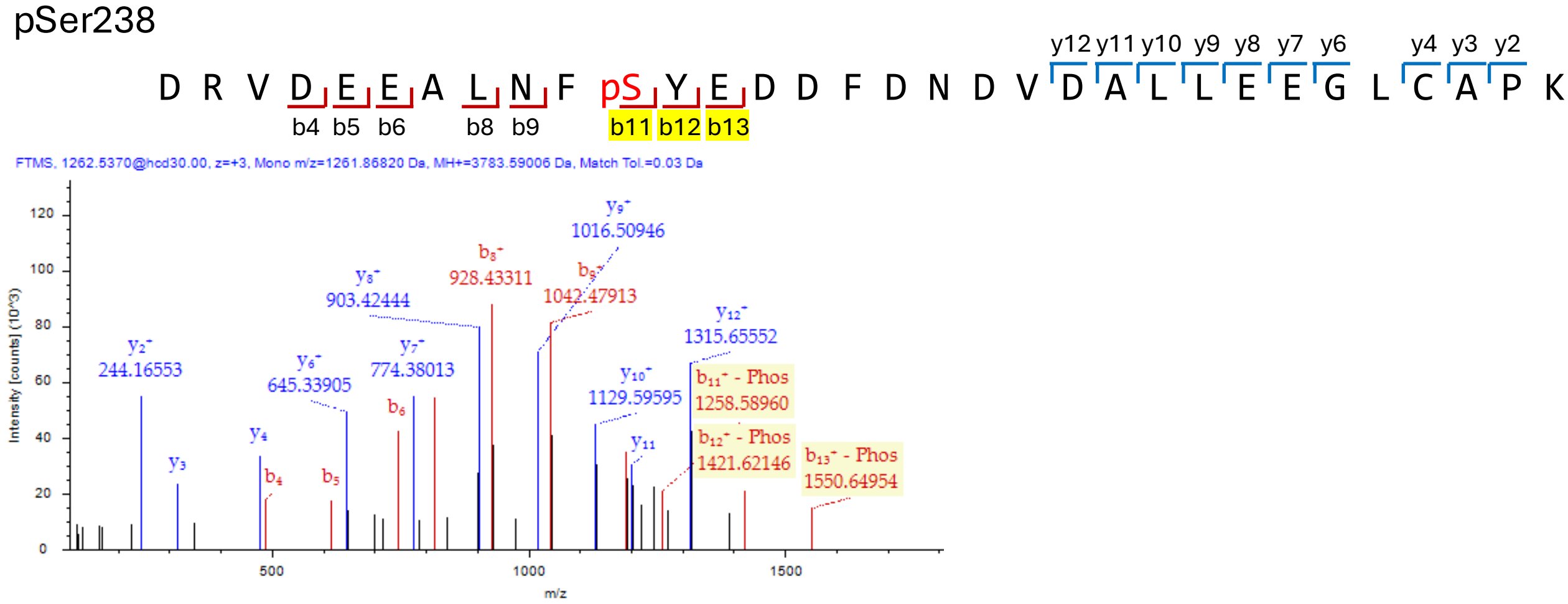

pSer271

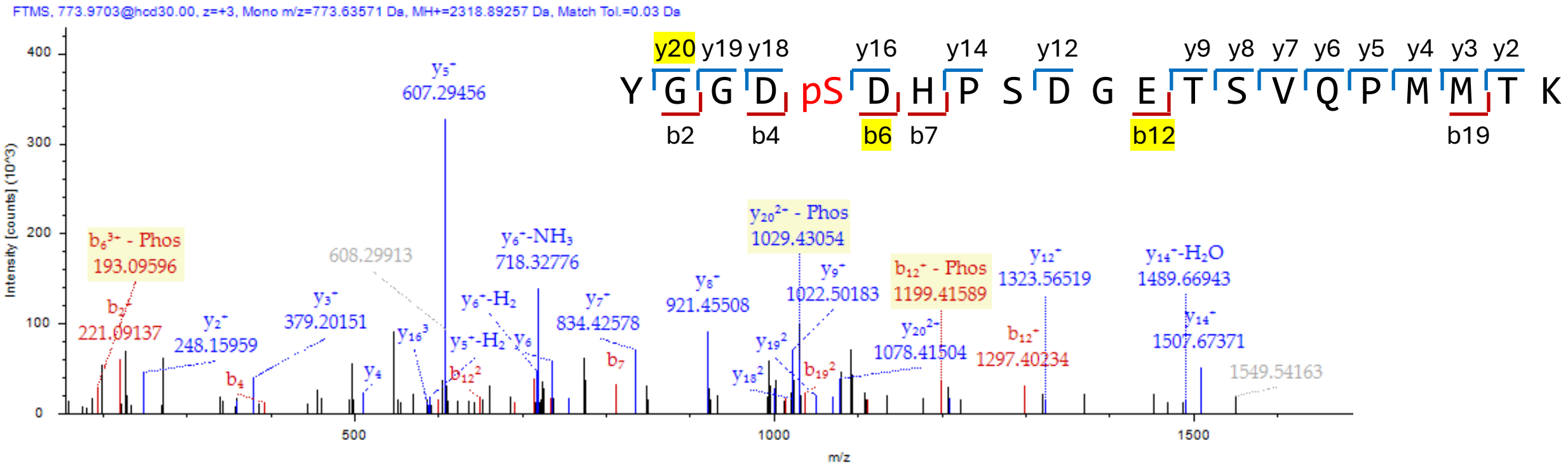

pSer275

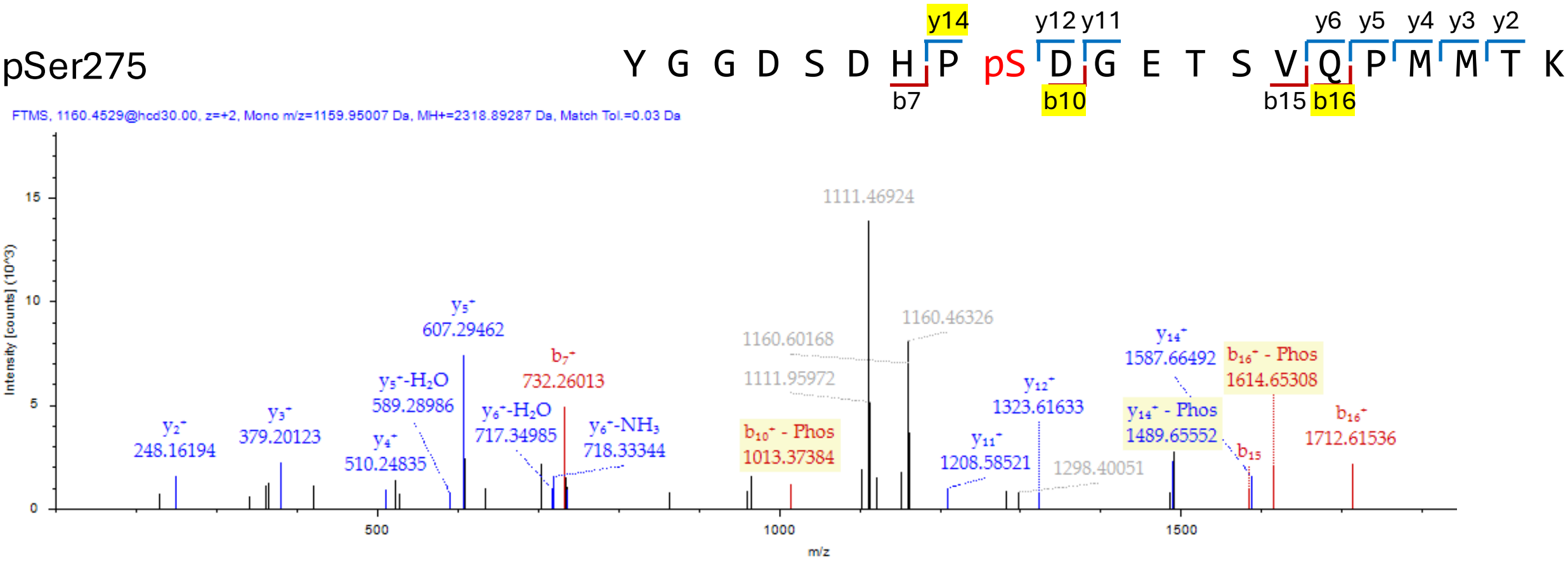

pSer373 and pSer377

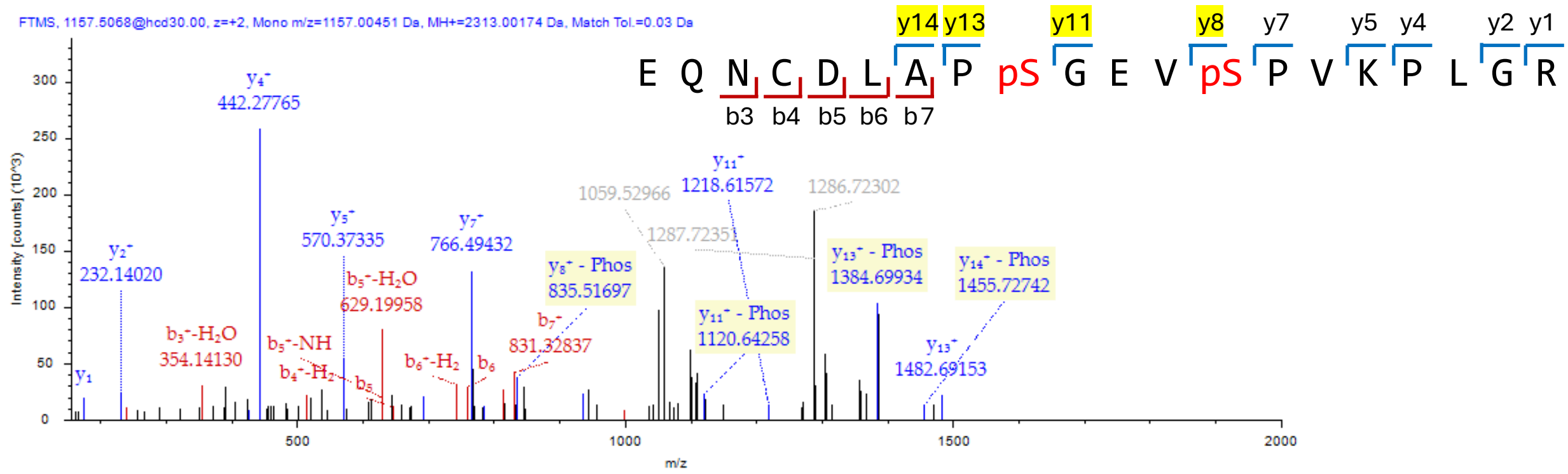

pSer434

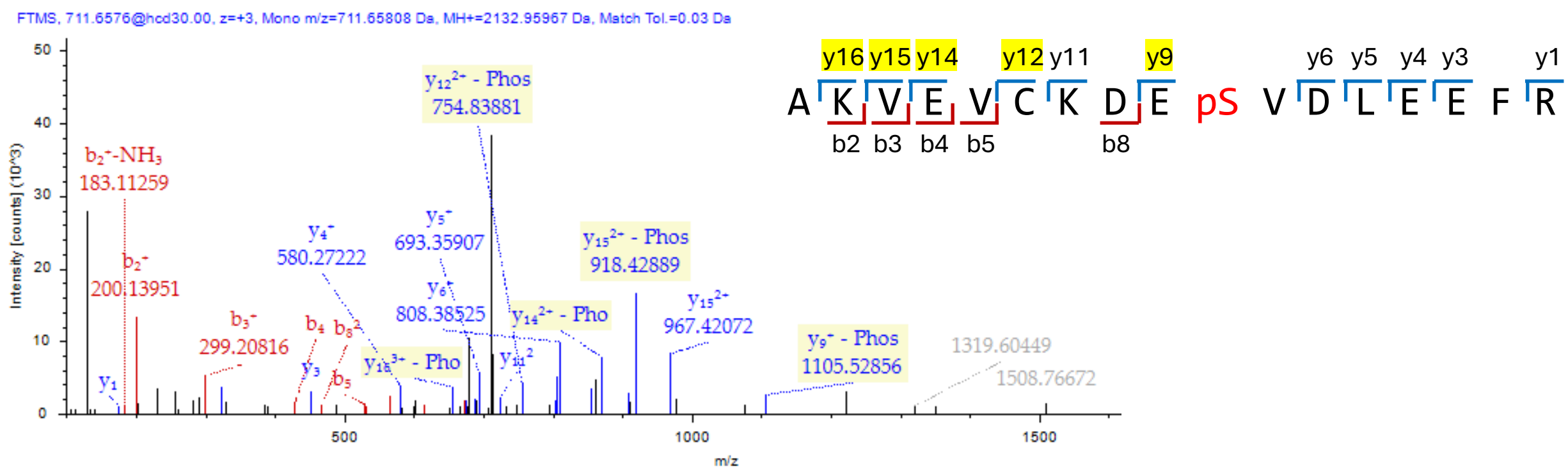

pSer619

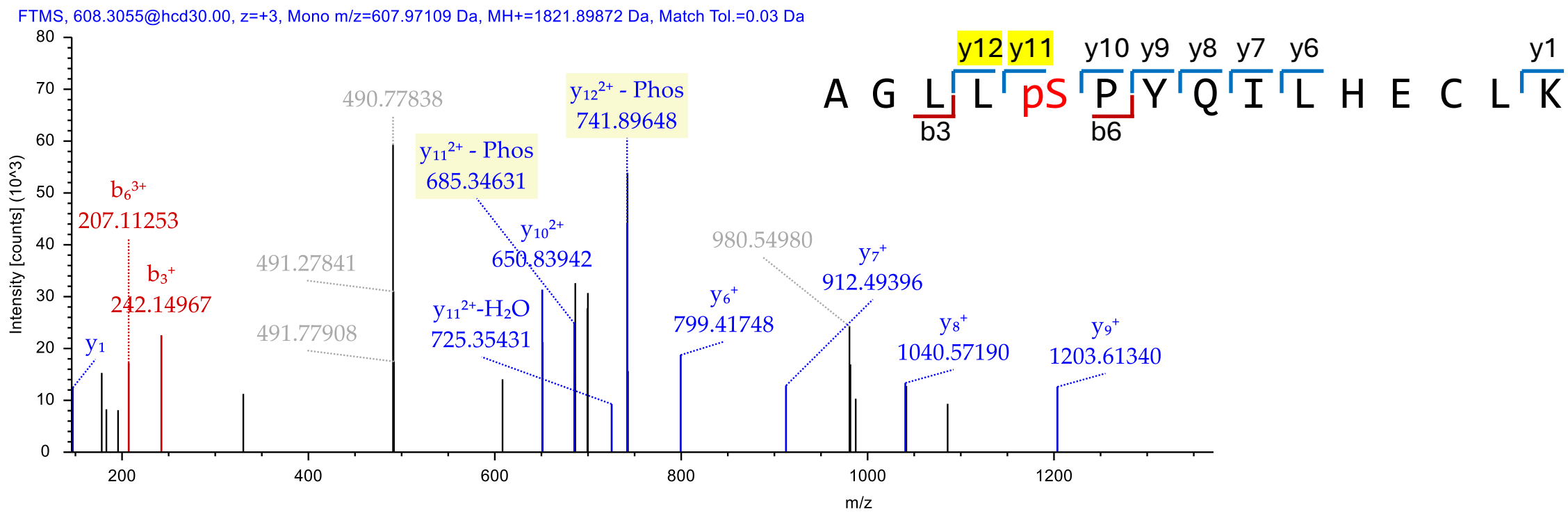

pSer714 and pSer/Thr/Tyr

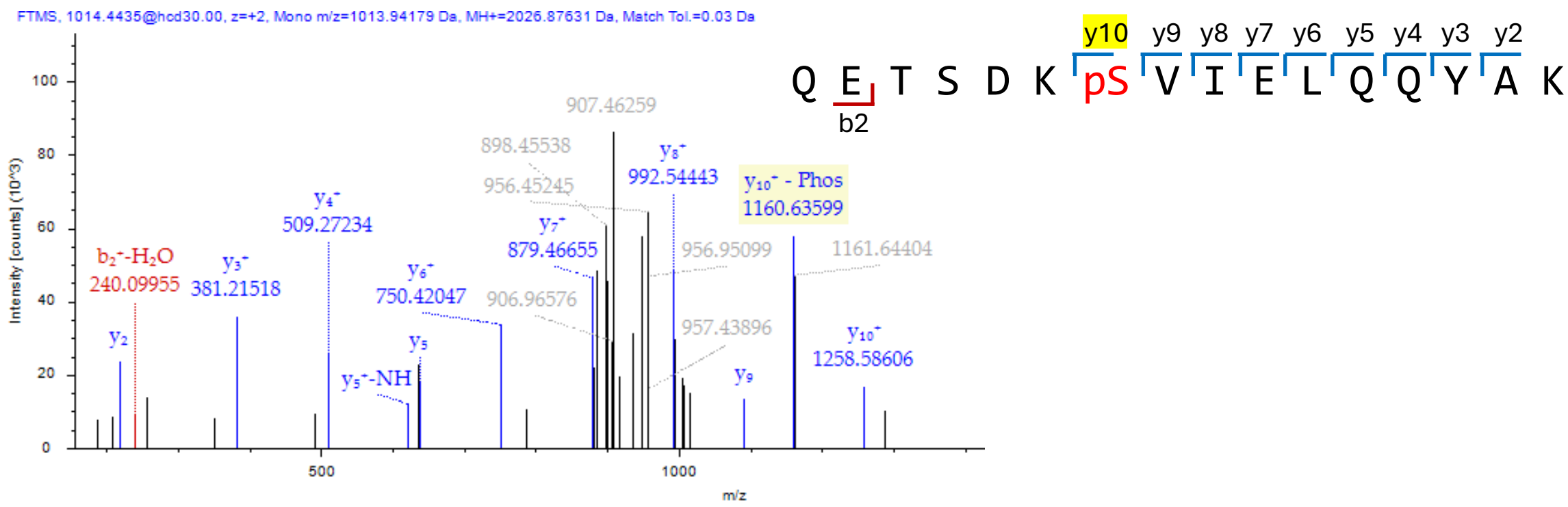

Supplementary Figure 2. Representative fragment ion mass spectra for the identification of phosphorylated sites in endogenous murine DGCR8. Only the y- and b- ions with the highest abundances are labelled. Phosphorylated ions are highlighted in yellow. Fragment ion mass spectra identifying pSer8, pSer95, pSer109, pSer238, pSer271, pSer275, pSer434, and pSer619 were obtained from mono-phosphorylated precursor ions, while spectra identifying pSer373/pSer377 and pSer714 were obtained from di-phosphorylated precursor ions.

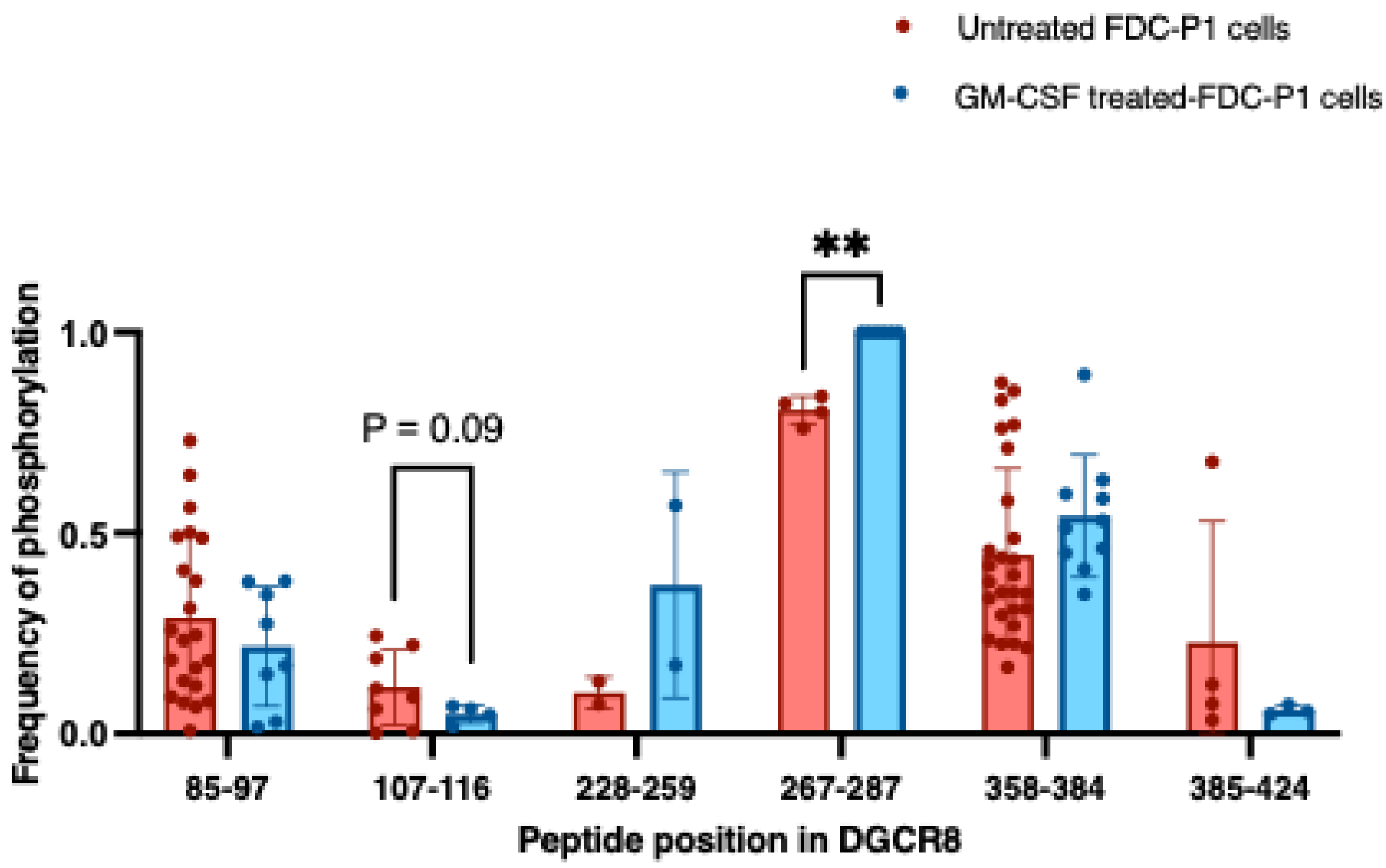

Supplementary Figure 3. DGCR8 is differentially phosphorylated in FDC-P1 cells upon myeloid differentiation. Phosphorylation frequency of endogenous DGCR8 trypsin-digested peptides extracted from 16 independent IPs of undifferentiated FDC-P1 cells and 5 IPs GM-CSF-differentiated cells. A total of 42 and 10 DGCR8 protein samples were analyzed, respectively. Error bars represent mean ± SD; ** $P < 0.01$; unpaired t-test with Welch's correction.

pThr272

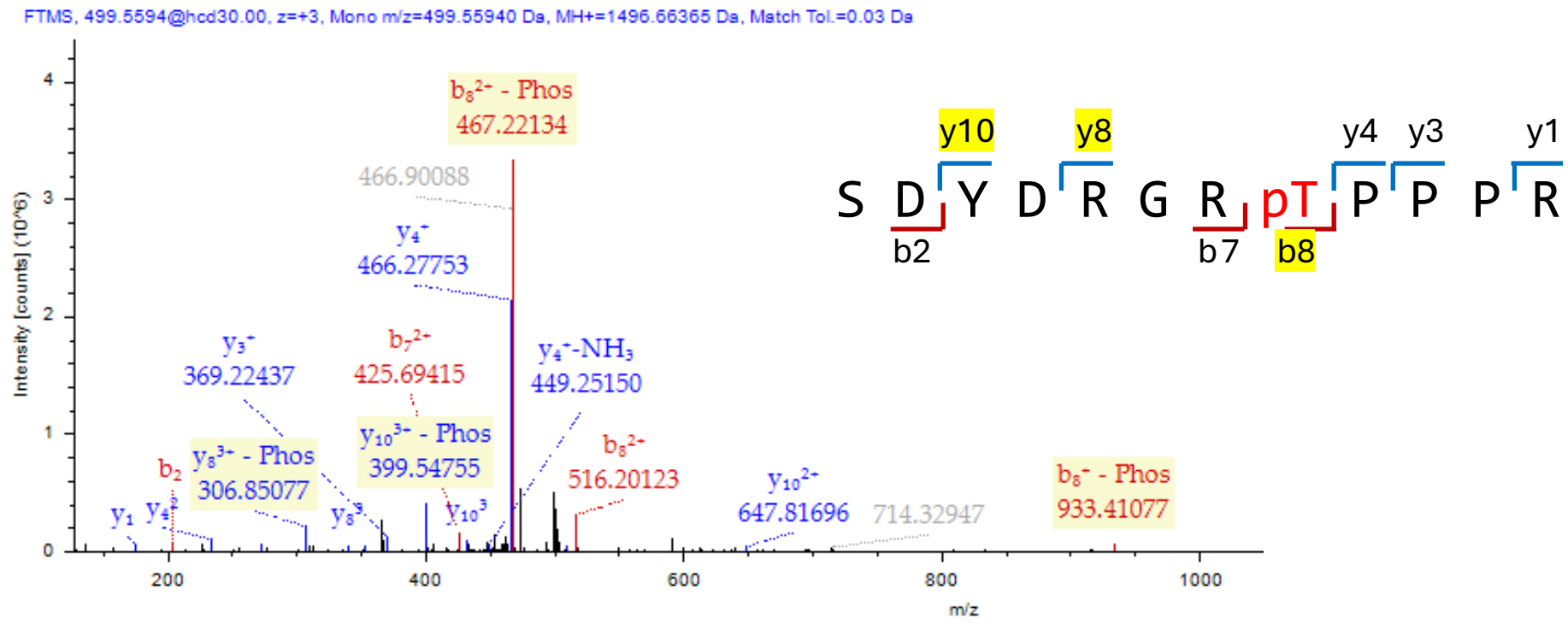

pSer354 and pSer356

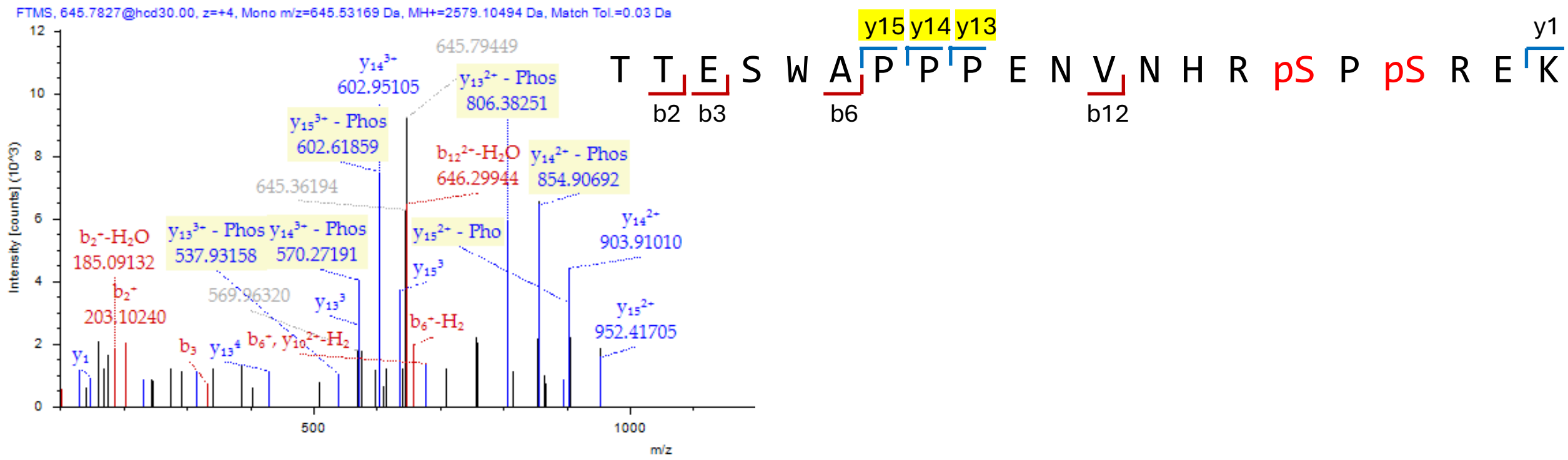

pSer372

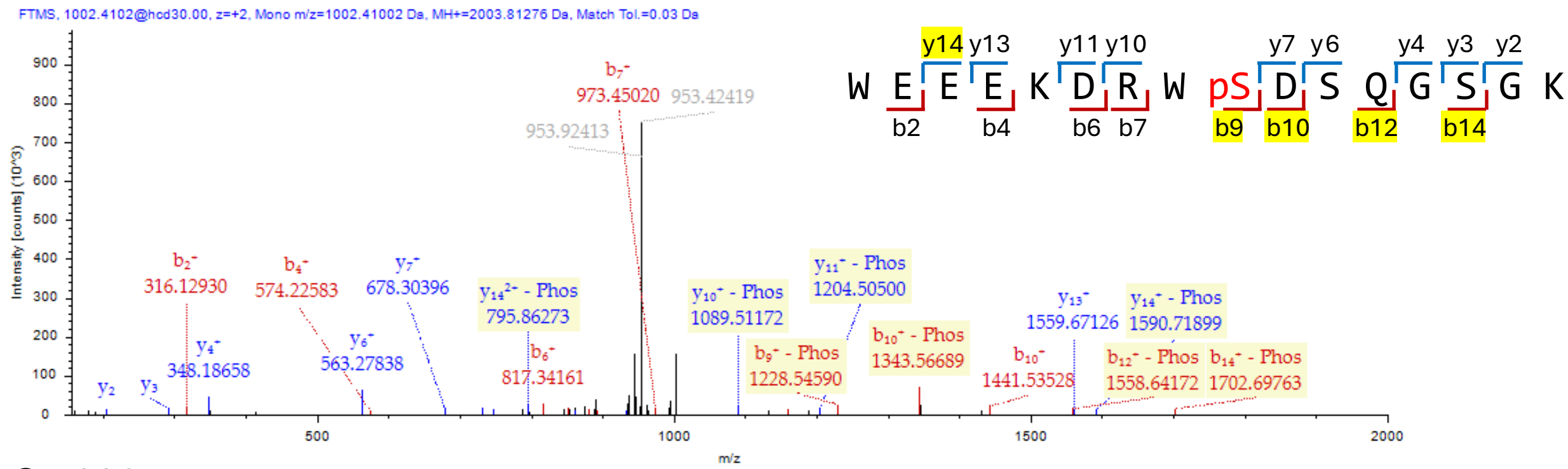

pSer806

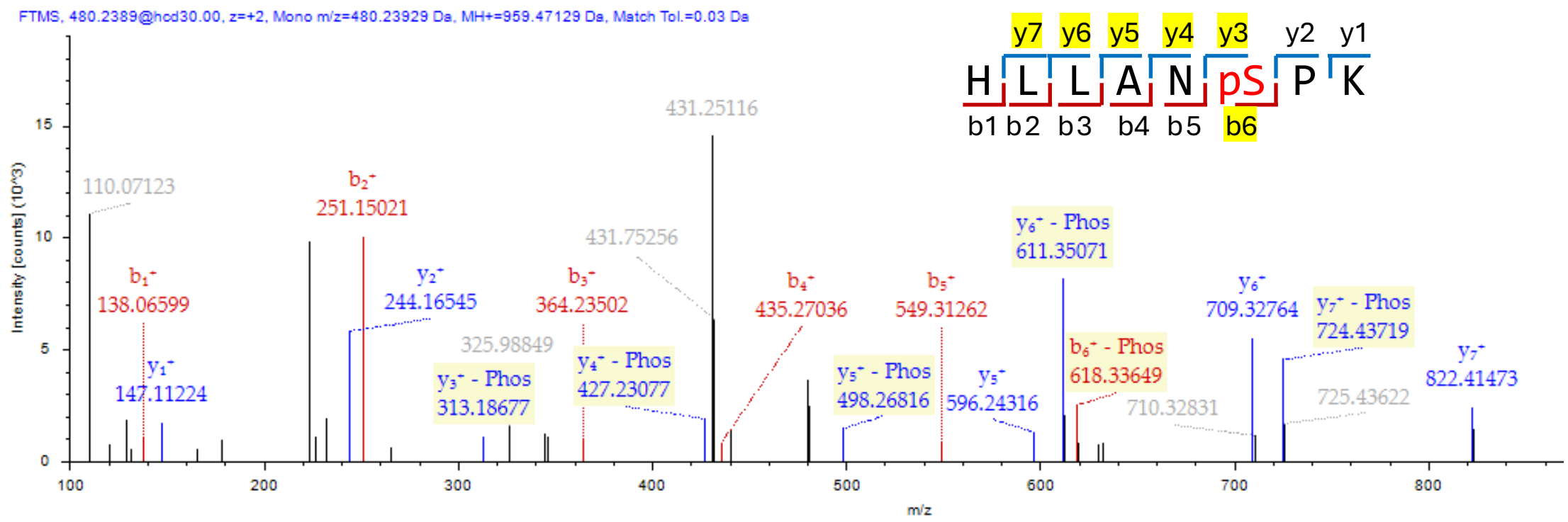

Supplementary Figure 4. Representative fragment ion mass spectra for the identification of phosphorylated sites in endogenous murine DROSHA. Only the y- and b- ions with the highest abundances are labelled. The phosphorylated ions are highlighted in yellow. Fragment ion mass spectra identifying pThr272, pSer372, and pSer806 were obtained from mono-phosphorylated precursor ions, while the spectrum identifying pSer354/pSer356 was obtained from a di-phosphorylated precursor ion.

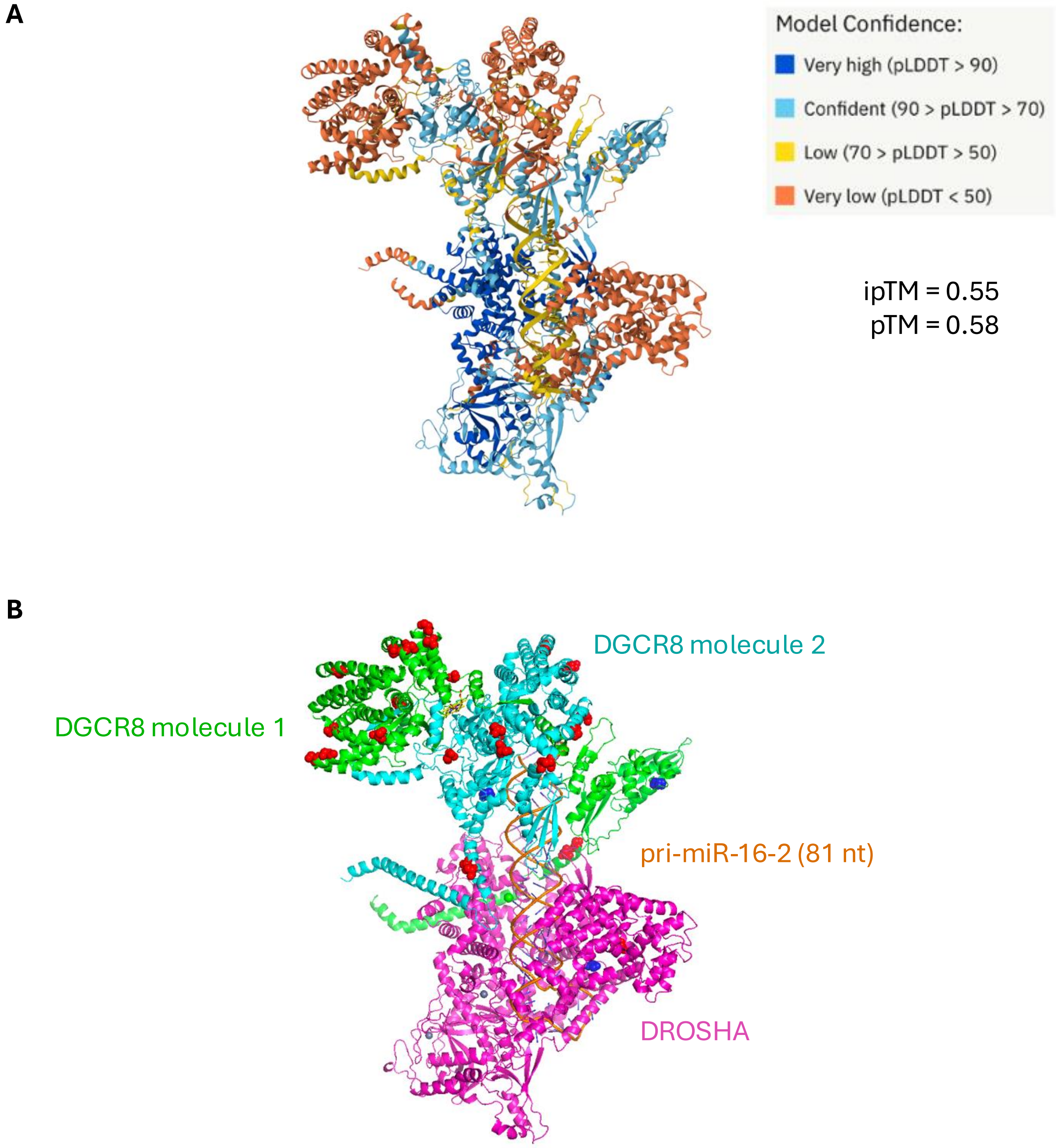

Supplementary Figure 5. AlphaFold3 prediction the Microprocessor structure. The input components include two molecules of Mus musculus DGCR8 (UniProt ID: Q9EQM6), one molecule of Mus musculus DROSHA (UniProt ID: Q5HZJ0), one heme molecule, two zinc ions, one calcium ion, and one pri-miR-16-2 (81-nt, extracted from PDB ID: 6V5B). The model was generated using AlphaFold3 with PDB templates available up to 30/09/2021. **(A)** Model coloured according to model confidence. An ipTM < 0.6 suggests low confidence in the relative positioning of the components within the complex. A pTM > 0.5 indicates that the overall fold of the complex is likely reliable. **(B)** Model coloured by component. Red and blue spheres indicate the identified phosphorylation sites located in the predicted intrinsically disordered and ordered regions, respectively.

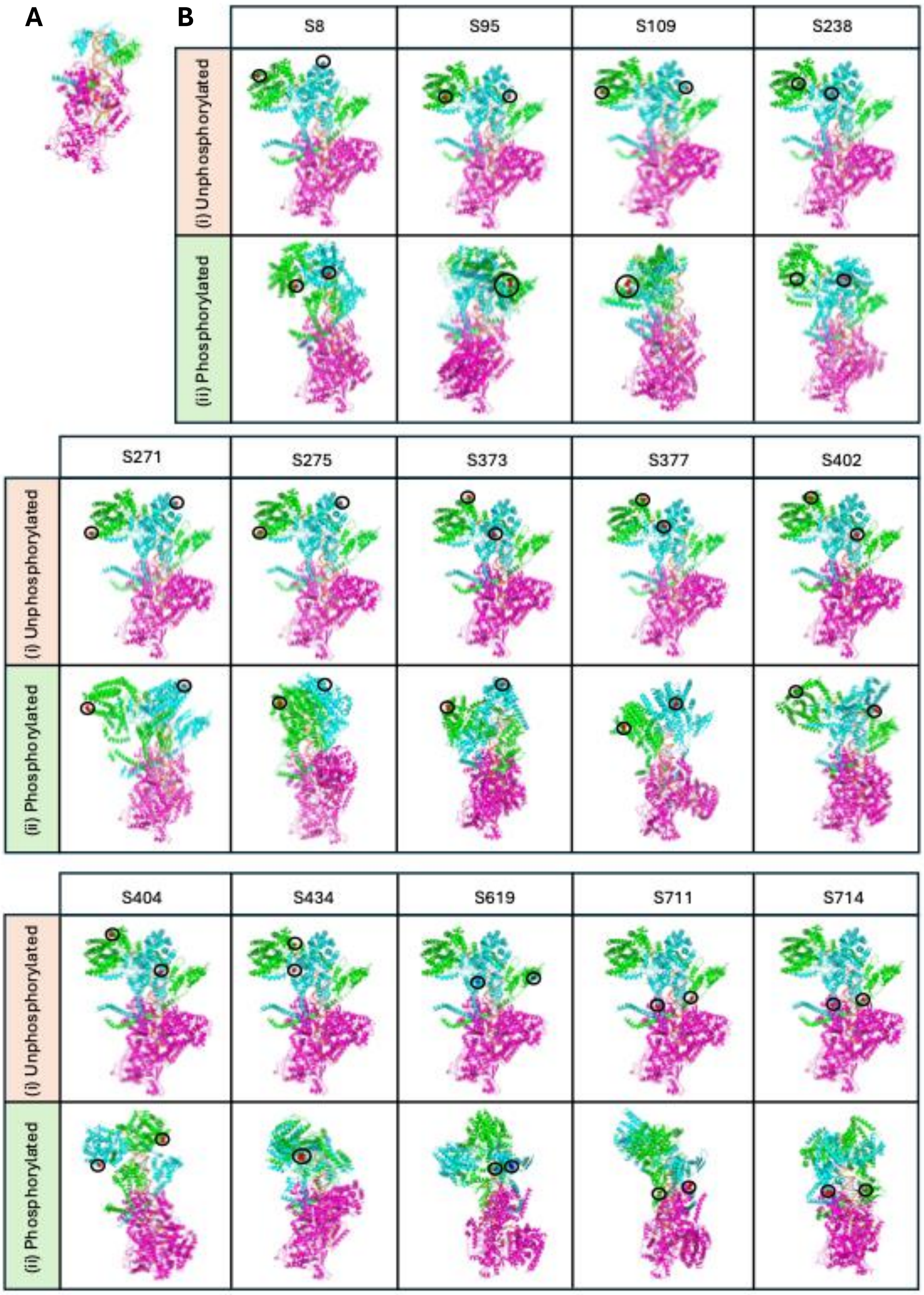

Supplementary Figure 6. Predicted structural models of the Microprocessor complex with DGCR8 phosphorylated at different residues. (A) Previously reported cryo-EM structure of the Microprocessor complex (PDB ID: 6V5B). **(B)** AlphaFold3-predicted models of the Microprocessor complex including (i) unphosphorylated and (ii) DGCR8 phosphorylated at individual sites identified in Chapter 3. The model was generated using PDB templates with cut-off date of 30/09/2021. The models were structurally aligned to enable comparison. Sites of interest are indicated with black circles. Red and blue spheres indicate the phosphorylation sites located in the predicted intrinsically disordered and ordered regions, respectively. All the predicted models have pTM scores over 0.5, except for those phosphorylated at S373 and S434. All the predicted models have ipTM scores lower than 0.6. Images generated using PyMOL (version 3.1.7.2). The two DGCR8 molecules are coloured green and cyan, DROSHA is magenta, and pri-miR-16-2 is orange.

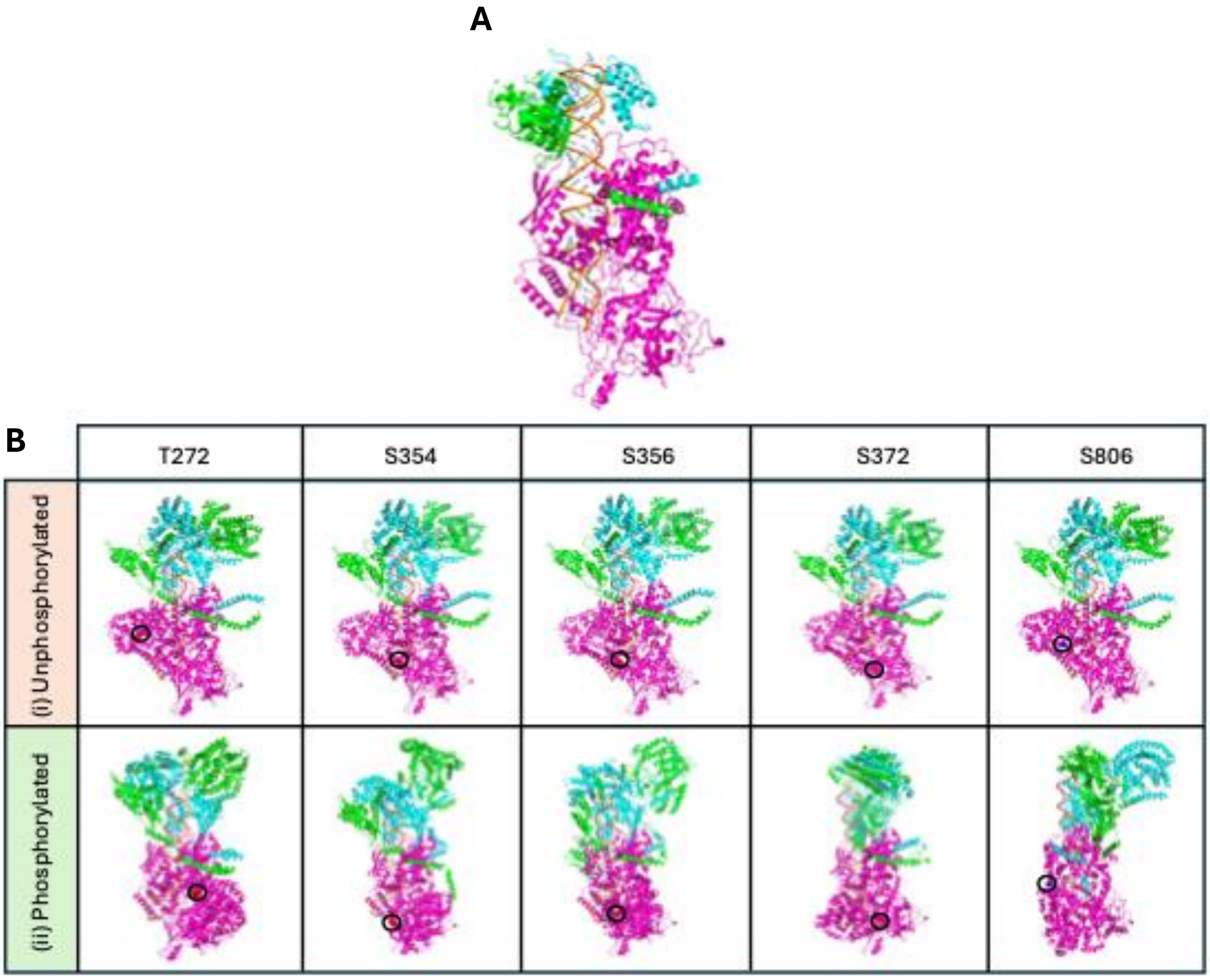

Supplementary Figure 7. Predicted structural models of the Microprocessor complex with DROSHA phosphorylated at different residues. (A) Previously reported cryo-EM structure of the Microprocessor complex (PDB ID: 6V5B). **(B)** AlphaFold3-predicted models of the Microprocessor complex including (i) unphosphorylated and (ii) DROSHA phosphorylated at individual sites identified in Chapter 3. The model was generated using PDB templates with cut-off date of 30/09/2021. The models were structurally aligned to enable comparison. Sites of interest are indicated with black circles. Red and blue spheres indicate the phosphorylation sites located in the predicted intrinsically disordered and ordered regions, respectively. All the predicted models have pTM scores over 0.5 and ipTM scores lower than 0.6. Images generated using PyMOL (version 3.1.7.2). The two DGCR8 molecules are coloured green and cyan, DROSHA is magenta, and pri-miR-16-2 is orange.

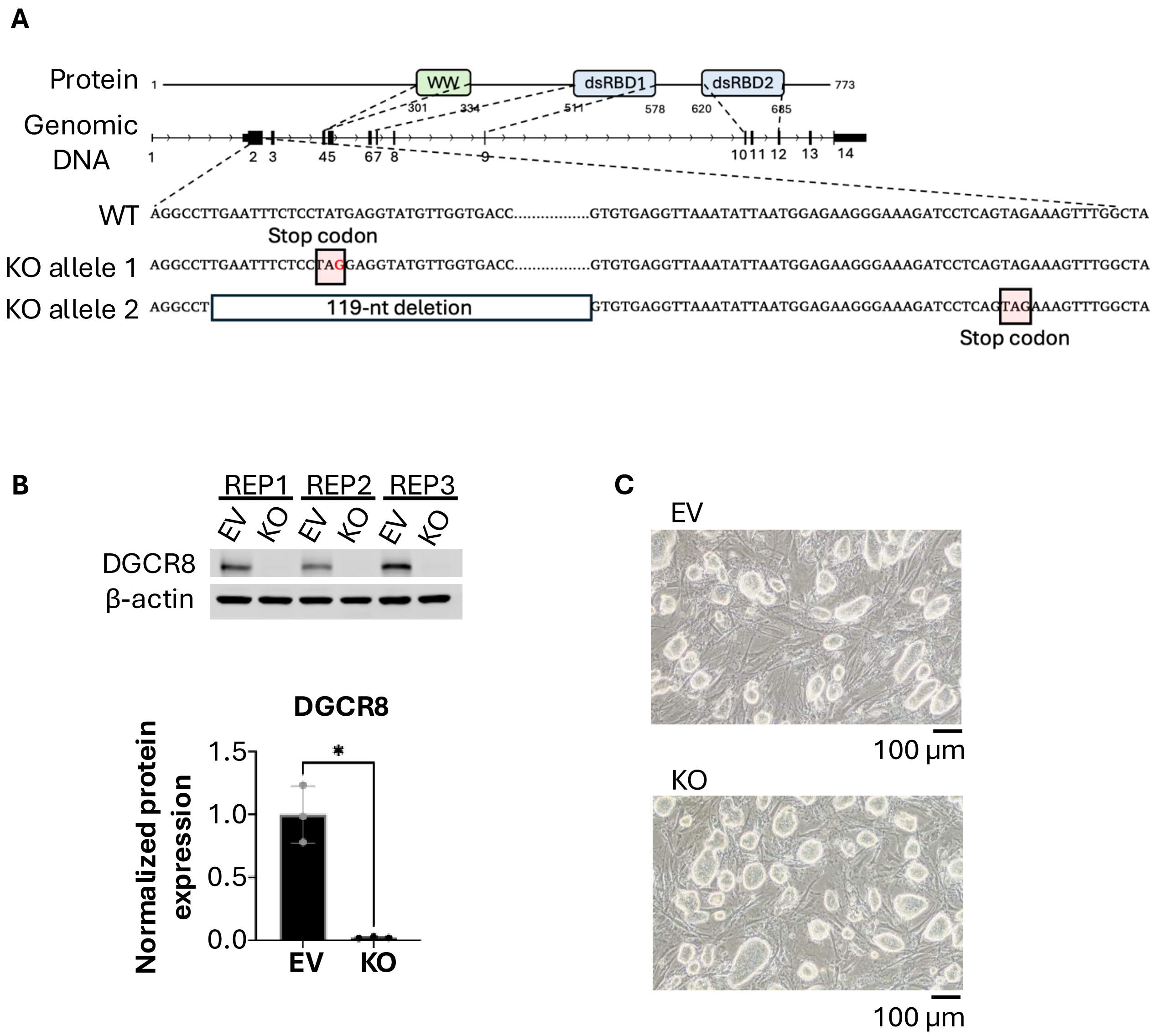

Supplementary Figure 8. Characterization of DGCR8 KO mESCs. (A) Sequences of the two DGCR8 alleles in DGCR8 KO E14 mESCs determined by Sanger sequencing. **(B)** Immunoblot of DGCR8 in DGCR8 empty vector (EV) mESCs and DGCR8 KO mESCs. Quantification of DGCR8 normalized to β-actin from three independent cell cultures. Error bars represent mean ± SD; * $P < 0.05$; unpaired t-test with Welch's correction. **(C)** Representative bright field images of DGCR8 EV mESCs and DGCR8 KO mESCs.

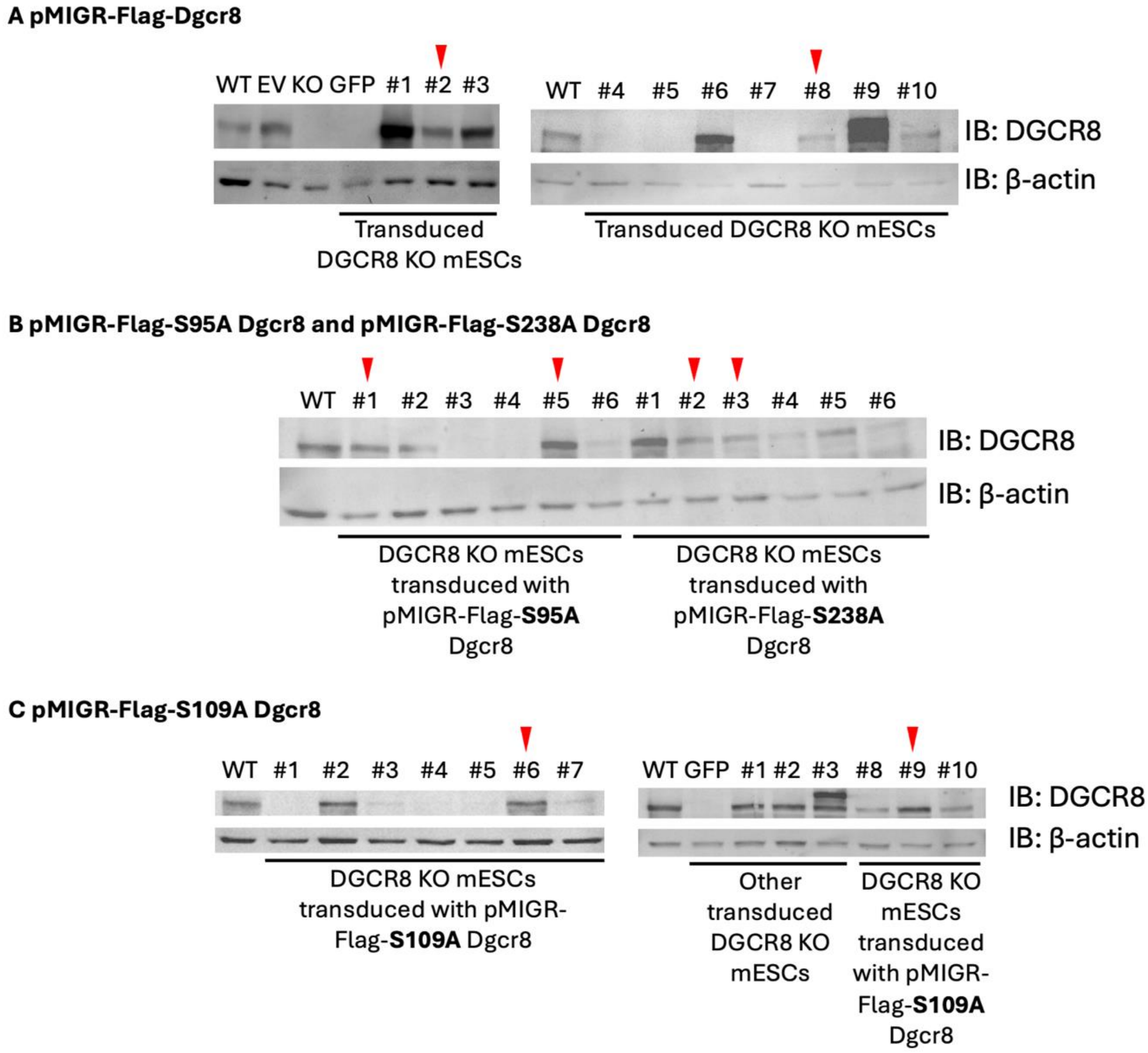

Supplementary Figure 9. Selection of DGCR8-re-expressing mESCs. Immunoblot analysis of DGCR8 and β-actin in WT mESCs, lentiCRISPRv2-transduced mESCs (EV), DGCR8 KO mESCs (KO) and DGCR8 KO mESCs transduced with the empty MSVC-IRES-GFP vector (pMIGR), **(A)** pMIGR-Flag-Dgcr8, **(B)** pMIGR-Flag-S95A Dgcr8 or pMIGR-Flag-S238A Dgcr8, or **(C)** pMIGR-Flag-S109A Dgcr8. Clones selected for subsequent experiments are indicated by red arrows.

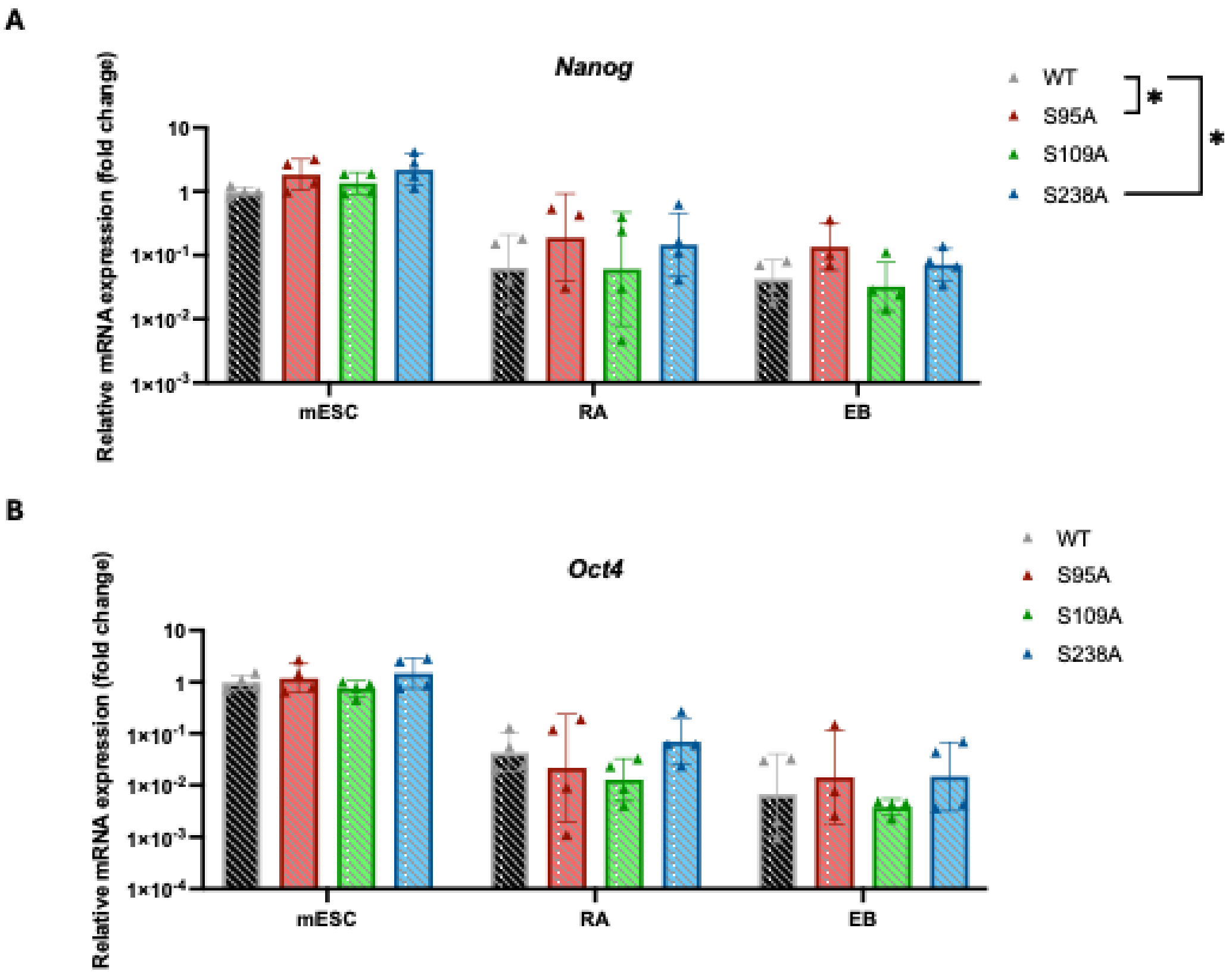

Supplementary Figure 10. Pluripotency marker expression in DGCR8-transduced mESCs before and after neuronal and spontaneous differentiation. DGCR8 KO mESCs transduced with WT DGCR8, S95A DGCR8, S109A DGCR8, or S238A DGCR8 were subjected to neuronal differentiation (RA) or embryoid body formation followed by spontaneous differentiation (EB), then analysed by quantitative RT-PCR for the pluripotency markers **(A)** *Nanog* and **(B)** *Oct4*. mRNA expression levels were normalised to *Actb* mRNA and are presented as relative to the mean expression of WT DGCR8-transduced DGCR8 KO mESCs. Error bars represent geometric mean ± geometric SD; * $P < 0.05$; two-way ANOVA with Tukey's correction. Only statistically significant comparisons between genotypes are indicated.

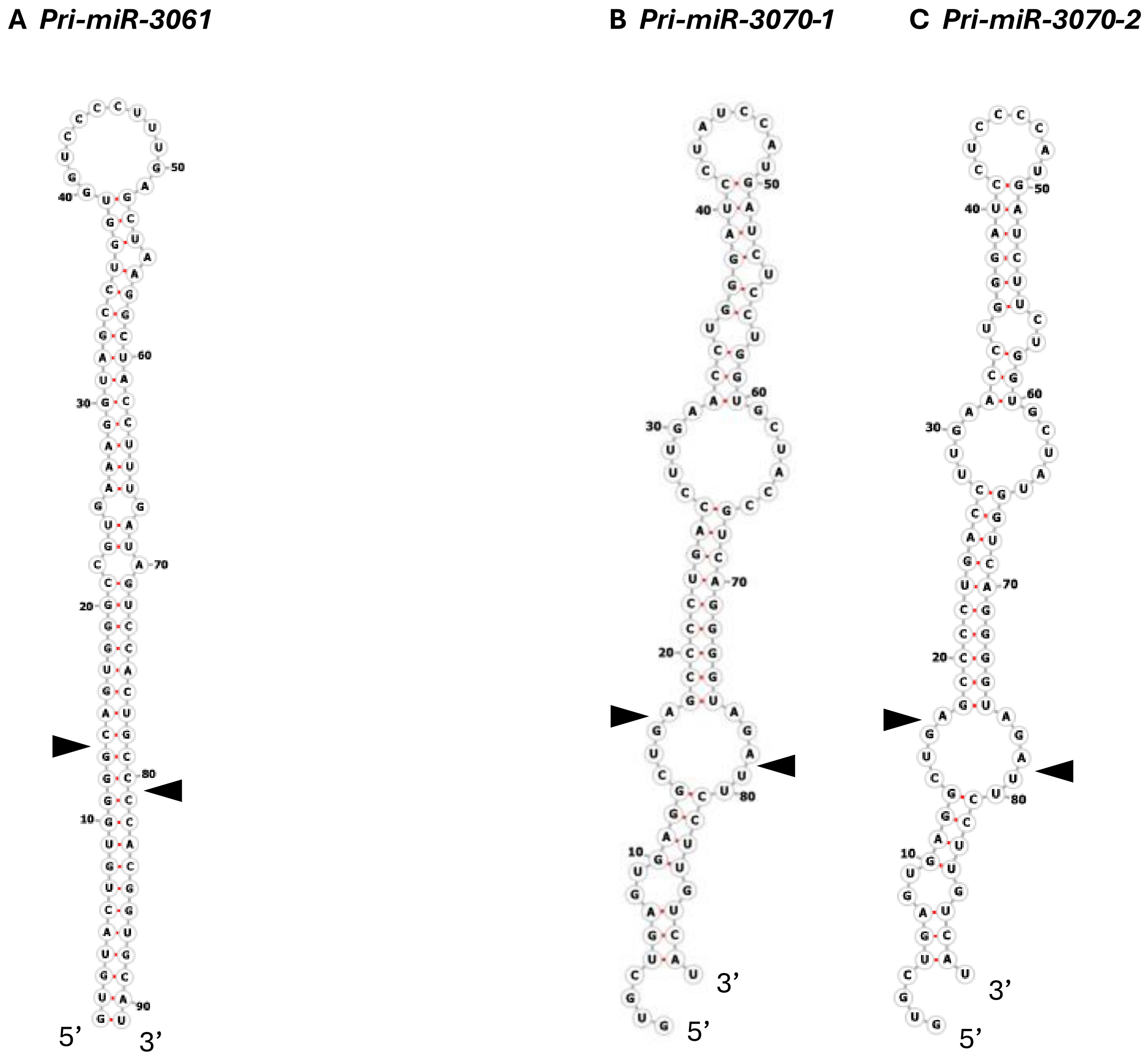

Supplementary Figure 11. Upregulated miRNAs in S95A DGCR8 mESCs exhibit atypical stem-loop structures in their primary transcripts. RNAfold minimal free energy (MFE) structural prediction of murine **(A)** *pri-miR-3061*, **(B)** *pri-miR-3070-1*, and **(C)** *pri-miR-3070-2* transcripts visualized using forna. The putative cleavage sites by DROSHA are indicated by black arrows.

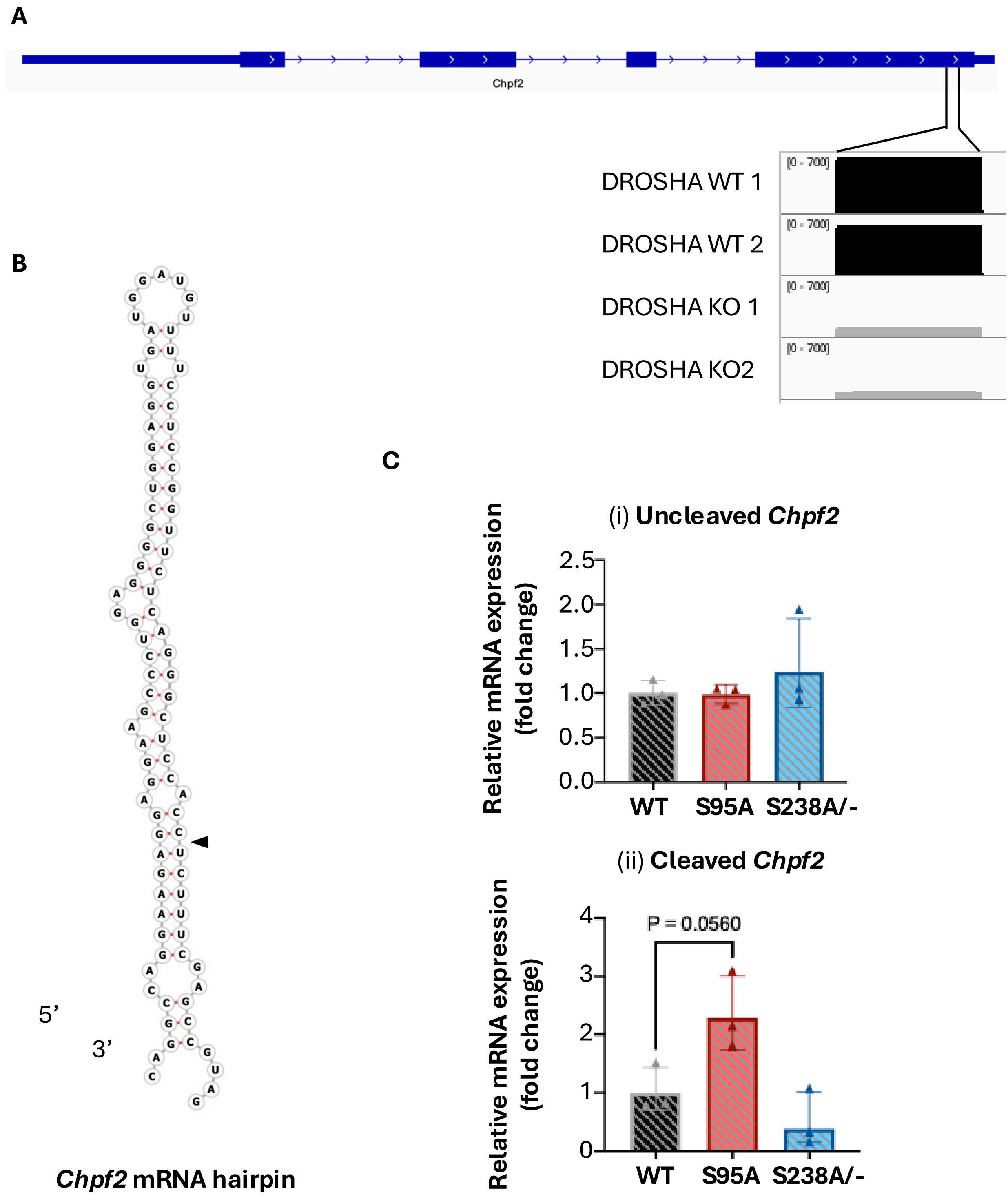

Supplementary Figure 12. *Chpf2* mRNA is a cleavage target of the Microprocessor complex. (A) Degradome-sequencing reads piling up in the *Chpf2* mRNA coding region. Data obtained from GEO database GSE228299 and visualized using IGV (Version 2.19.7). **(B)** RNAfold minimal free energy (MFE) structural prediction of the sequence surrounding the cleavage site in the *Chpf2* mRNA. The structure was visualized using forna. The putative cleavage site is indicated with a black arrow. **(C)** Quantitative RT-PCR analysis for (i) uncleaved and (ii) cleaved *Chpf2* mRNA levels in mutant mESCs. The mRNA expression levels were normalized to *Actb* mRNA and presented as relative to the mean expression of WT mESCs. Error bars represent geometric mean ± geometric SD. Unpaired t-test with Welch's correction.

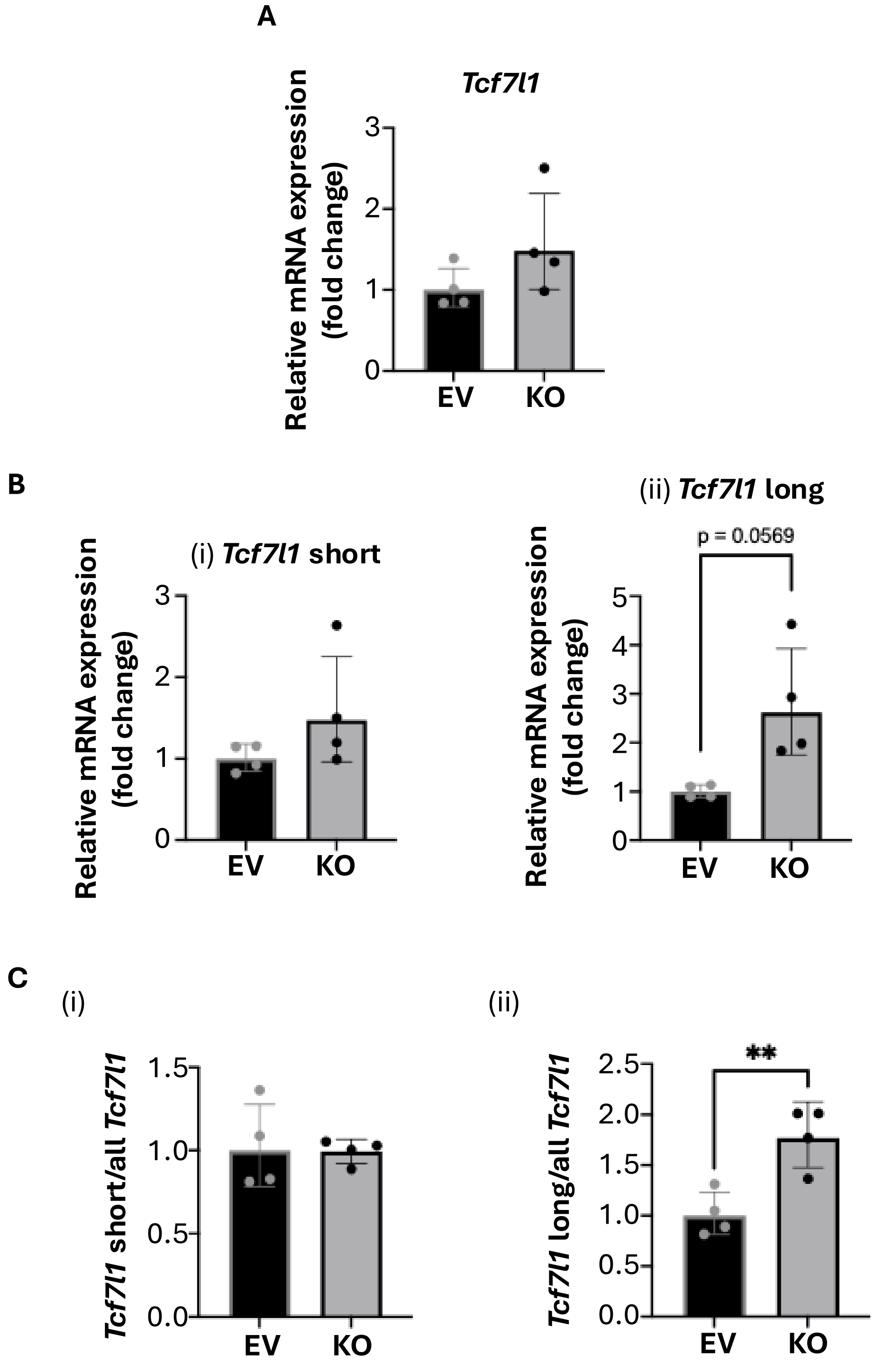

Supplementary Figure 13. DGCR8 deficiency shift *Tcf7l1* mRNA splicing towards the long isoform in mESCs. (A) Quantitative RT-PCR analysis for total *Tcf7l1* mRNA normalized to *Actb* mRNA. **(B)** Quantitative RT-PCR analysis for (i) *Tcf7l1* short and (ii) *Tcf7l1* long isoform mRNAs normalized to *Actb* mRNA. **(C)** Quantitative RT-PCR analysis for (i) *Tcf7l1* short and (ii) *Tcf7l1* long isoform mRNAs normalized to total *Tcf7l1* mRNA. All the mRNA expression levels were relative to the mean expression of DGCR8 EV mESCs. Error bars represent geometric mean ± geometric SD; ** $P < 0.01$; unpaired t-test with Welch's correction.

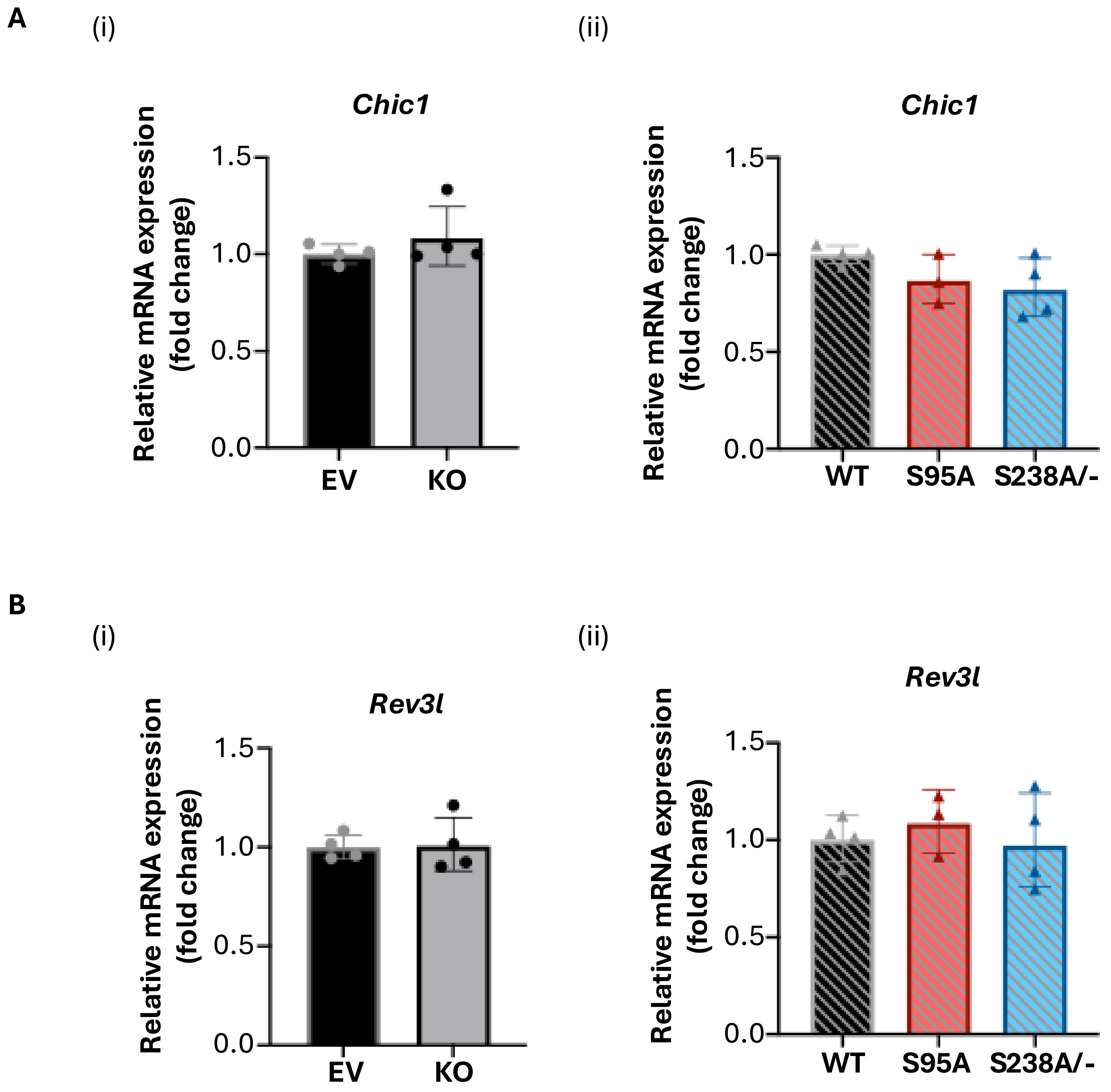

Supplementary Figure 14. Expression of poised genes are unaffected in DGCR8 mutant mESCs. Quantitative RT-PCR analysis for **(A)** *Chic1* and **(B)** *Rev3l* mRNA levels in (i) DGCR8 EV and DGCR8 KO mESCs, (ii) WT, S95A, S238A/- DGCR8 mESCs. mRNA expression levels were normalized to *Actb* mRNA and are presented relative to the mean expression of DGCR8 EV mESCs for panel (i) and WT mESCs for panel (ii). Error bars represent geometric mean ± geometric SD ; * $P < 0.05$; ** $P < 0.01$; Unpaired t-test with Welch's correction.

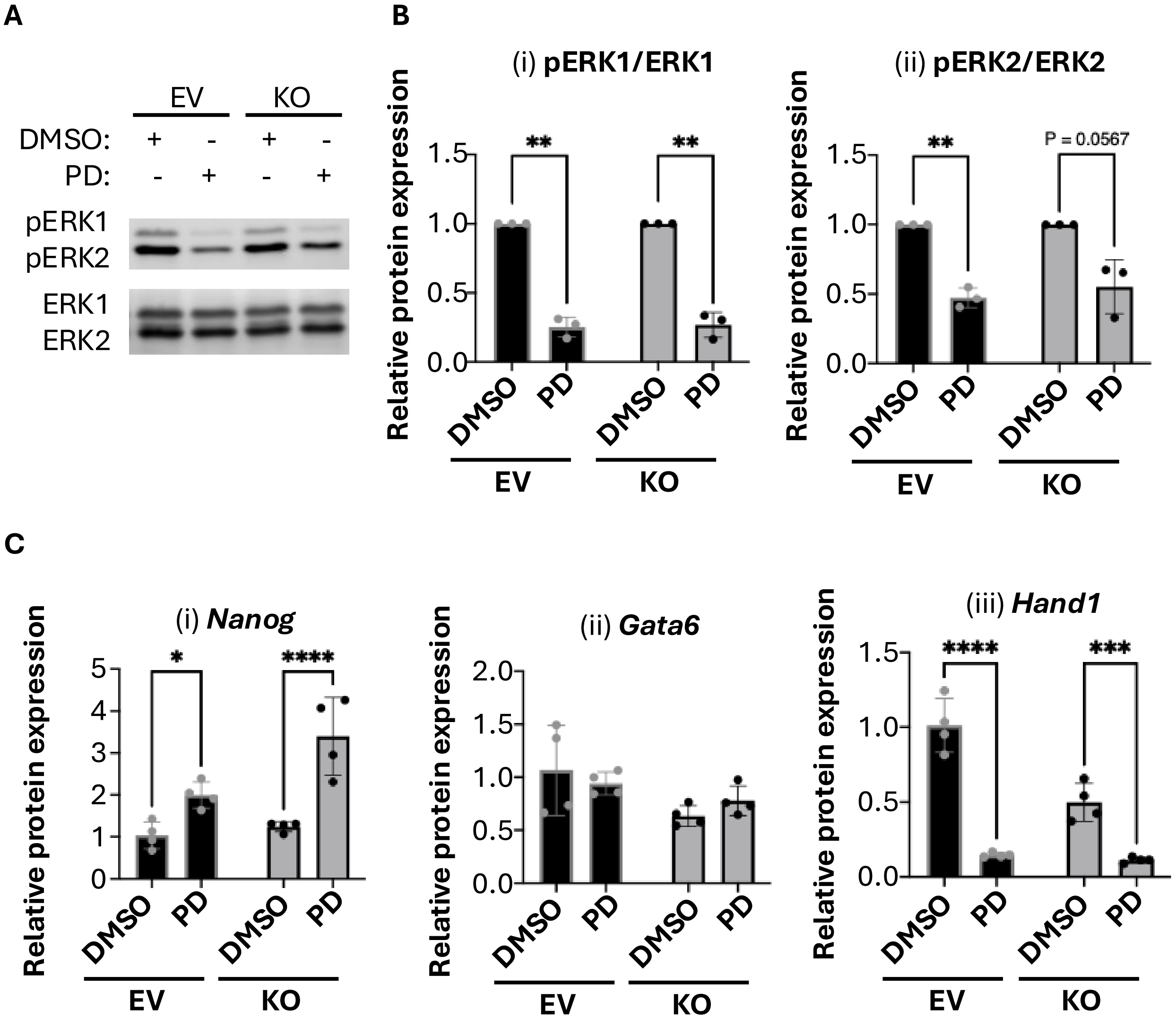

Supplementary Figure 15. MEK1/2 inhibition in mESCs. (A) Representative immunoblot for ERK1/2 and pERK1/2 protein levels in mESCs treated with either 0.01% DMSO or 1 µM PD0325901 (PD) for 24h. **(B)** Quantification of (i) pERK1 and (ii) pERK2 protein levels, normalized to ERK1 and ERK2, respectively, from three independent cell cultures. pERK1 and pERK2 expression levels in DGCR8 EV and DGCR8 KO mESCs are quantified relative to the corresponding DMSO-treated cultures. Error bars represent mean ± SD; ** $P < 0.01$; one-sample t-test against a hypothetical value of 1. **(C)** Quantitative RT-PCR analysis for (i) *Nanog*, (ii) *Gata6*, and (iii) *Hand1* mRNA levels in PD-treated mESCs. Expression levels were normalized to *Actb* mRNA and are presented relative to the mean expression of DGCR8 EV mESCs treated with DMSO. Error bars represent geometric mean ± geometric SD; * $P < 0.05$; *** $P < 0.001$; **** $P < 0.0001$; two-way ANOVA with uncorrected Fisher's LSD test. Only statistically significant comparisons between DMSO and PD0325901 treatments within each genotype are indicated.
